# Oxidative Dearomatization of PLP in Thiamin Pyrimidine Biosynthesis in *Candida albicans*

**DOI:** 10.1021/jacs.2c08560

**Published:** 2023-02-20

**Authors:** Anushree Mondal, Rung-Yi Lai, Dmytro Fedoseyenko, Nitai Giri, Tadhg P. Begley

**Affiliations:** Department of Chemistry, Texas A&M University, College Station, Texas77843, United States

## Abstract

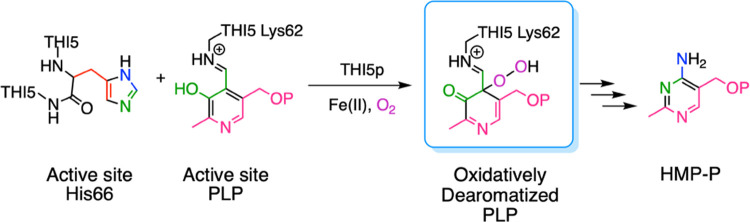

The yeast thiamin pyrimidine synthase THI5p catalyzes one of the most complex organic rearrangements found in primary metabolism. In this reaction, the active site His66 and PLP are converted to thiamin pyrimidine in the presence of Fe(II) and oxygen. The enzyme is a single-turnover enzyme. Here, we report the identification of an oxidatively dearomatized PLP intermediate. We utilize oxygen labeling studies, chemical-rescue-based partial reconstitution experiments, and chemical model studies to support this identification. In addition, we also identify and characterize three shunt products derived from the oxidatively dearomatized PLP.

## Introduction

**Figure 1:**
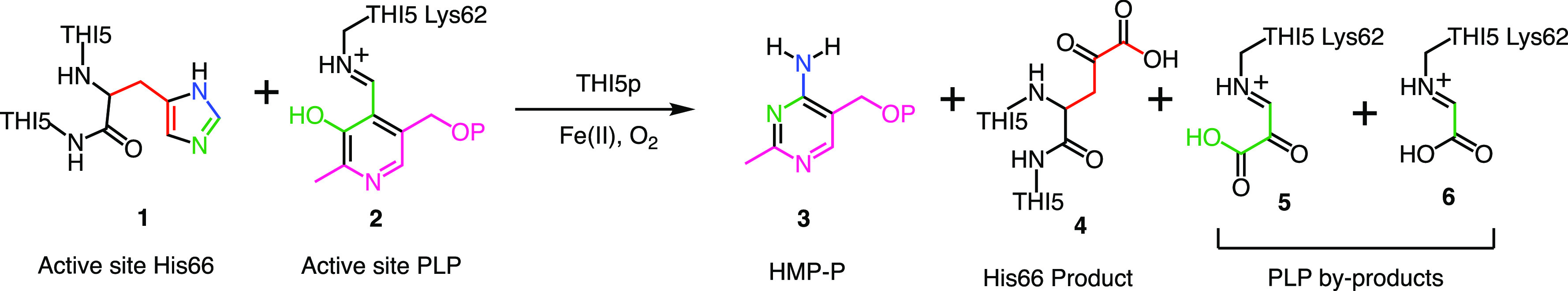
THI5p-catalyzed reaction showing the reaction products (**3**, **4**, **5**, and **6**).

**Figure 2:**
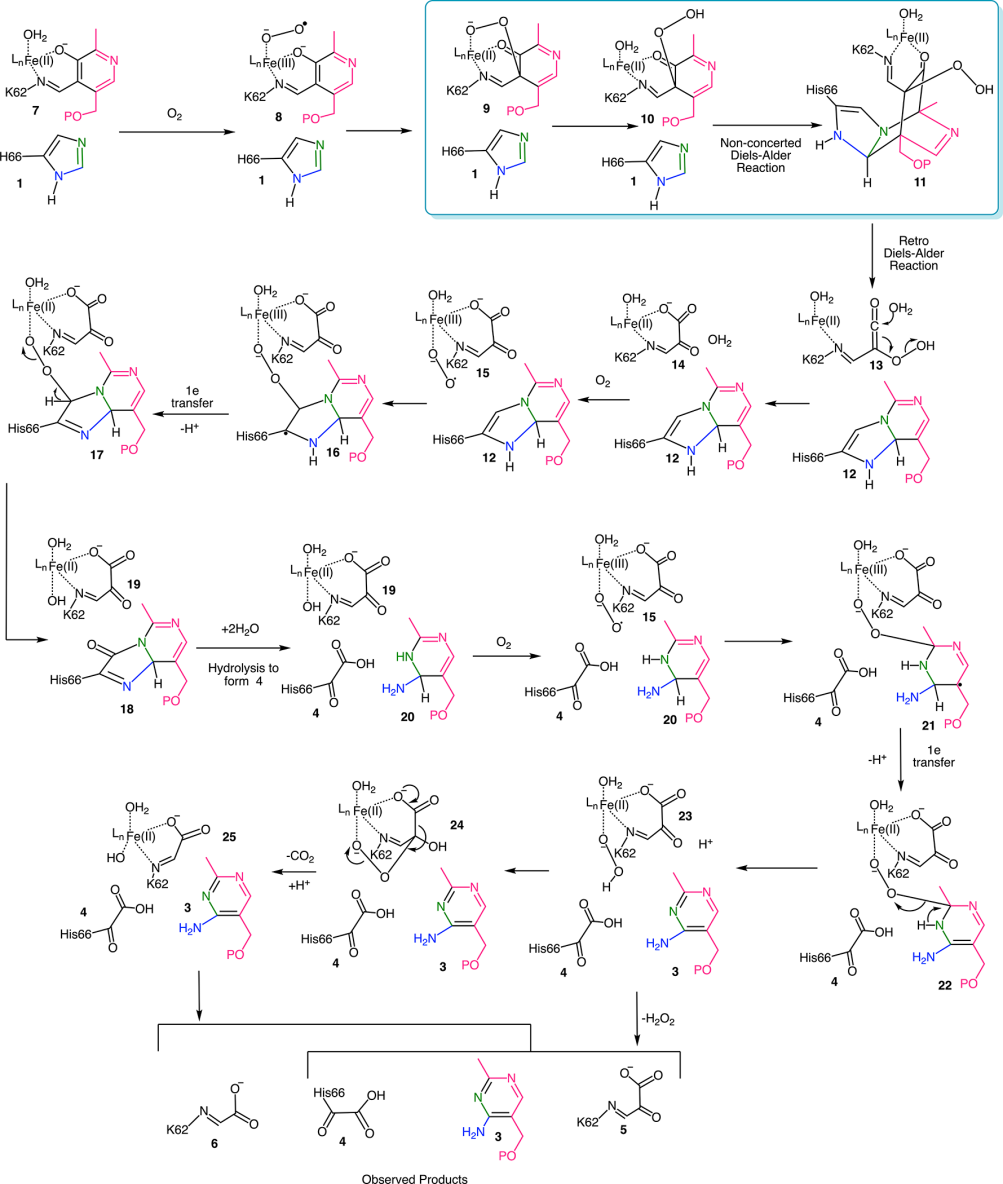
Current mechanistic proposal for the THI5p-catalyzed reaction. The conversion of **9** + **1** to **11** is the focus of this paper.

The microbial biosynthesis of thiamin pyrophosphate involves the coupling of the thiazole and pyrimidine heterocycles.^1−5^ In yeast, the thiazole synthase (THI4p) and the pyrimidine synthase (THI5p) both use enzyme-derived atoms for product formation and are single-turnover enzymes.^6−8^ While the mechanism of the thiazole synthase is relatively well-understood, mechanistic understanding of the pyrimidine synthase is still at an early stage.^7,9−13^ This enzyme uses the active site His66 (**1**) and the PLP imine (**2**) with Lys62 to form the thiamin pyrimidine (HMP-P, **3**). The reaction requires Fe(II) and oxygen (Figure 1).^6,8^ A mechanistic hypothesis for this complex reaction is shown in Figure 2.^8^ In this mechanism, PLP has been activated for a Diels–Alder reaction^14−16^ with the imidazole of His66 by an oxidative dearomatization to azadiene **10**. A retro-Diels–Alder reaction generates the heterocycle **12**, which undergoes iterative oxidation/hydrolysis reactions ultimately to give HMP-P (**3**) and the observed histidine and PLP byproducts (**4**–**6**). This mechanistic proposal is consistent with the structure of the enzyme–substrate complex, the identification of the PLP and His-derived byproducts, with labeling studies that establish the origin of all of the HMP-P nonhydrogen atoms, and with the Fe(II) and oxygen requirements of the reaction.^6,8,17−23^

THI5p is a challenging experimental system involving unprecedented PLP and histidine reactions. The turnover is low (∼0.3) because most of the enzyme (>60%) has undergone inactivation resulting from HMP-P (**3**) biosynthesis during overexpression.^8^ Therefore, a nontraditional strategy was required to elucidate its mechanism. In this paper, we describe a nonenzymatic model study to determine if dearomatized PLP can undergo Diels–Alder reactions and a set of experiments on the enzyme to probe for the oxidatively dearomatized PLP intermediate (**10**, Figure 2).

## Results and Discussion

### Oxygen Labeling Studies on the PLP-Derived Fragment **6**

The mechanism in Figure 2 predicts that the C2 ketone oxygen of **5** and both carboxylate oxygens of **6** are derived from molecular oxygen. This suggested that an ^18^O_2_ labeling experiment might provide a direct test of the oxidative dearomatization hypothesis.

**Figure 3:**
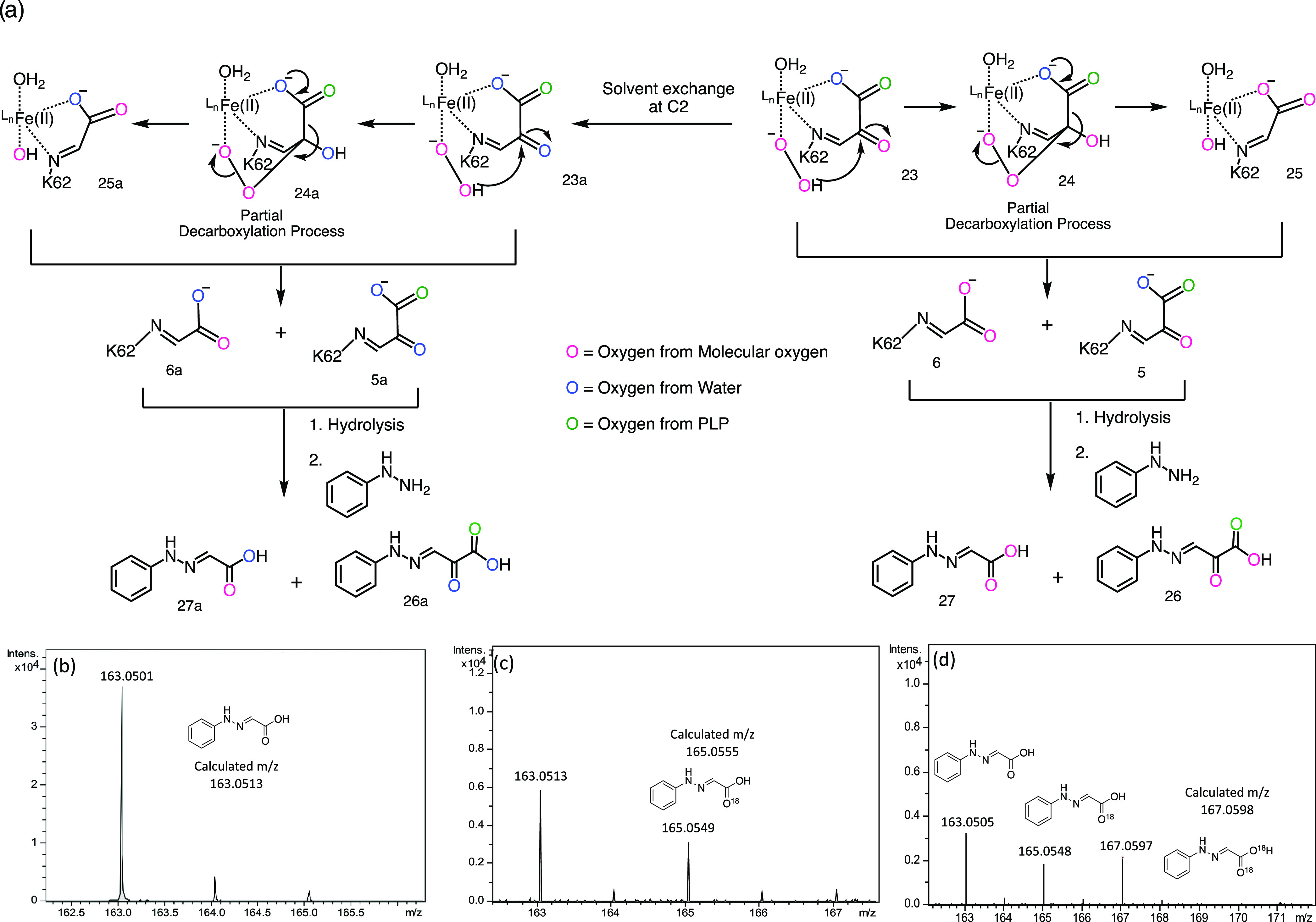
Oxygen labeling studies tracking molecular oxygen from peroxide **10** to hydrazone **27** and **27a**. (a) Scheme showing the oxygen labeling pattern (including the possibility for solvent exchange) for the PLP byproducts **5** and **6**. (b) MS of the glyoxal hydrazone formed under standard THI5p reaction conditions. (c) MS of the glyoxal hydrazone formed in [^18^O]-H_2_O/H_2_O (1:1).^18^ (d) MS spectrum of the glyoxal hydrazone formed in ^18^O_2_ shows M + 2 and M + 4 signals demonstrating that oxygen from azadiene **10** is retained in the glyoxal hydrazone **27**.

The exchange of the ketone oxygen in α-ketoacids with water potentially complicates tracking the peroxide oxygen of dearomatized PLP (**10**). This exchange would result in the replacement of the peroxide-derived oxygen in **14**, **15**, **19**, and **23** with buffer-derived oxygen. Ketone oxygen exchange during the conversion of **5** to the corresponding hydrazone **26** (Figure 3a) is also likely to occur making it impossible to track the peroxide oxygen from **10** to **26** (Figure 3a). Our analysis therefore focused on determining the origin of the carboxylate oxygens of **6** with the hope that restricted active site access to water would retard exchange from **14**, **15**, **19**, and **23** and that active site decarboxylation of **23** to form carboxylate **6** would retard exchange of the proposed peroxide-derived oxygen during hydrazone formation.

Our experimental protocol to determine the origin of the carboxylate oxygens of **6** is outlined in Figure 3a. The reaction was run under standard reaction conditions using H_2_^18^O or ^18^O_2_ and quenched by ultrafiltration. The imine products were derivatized with phenyl hydrazine and analyzed by MS (Figures 3a, S2, and S3).^24^

To evaluate the extent of oxygen exchange, the reaction was first run in H_2_O/H_2_^18^O buffer (1:1) using ^16^O_2_. The mass spectra of synthetic and enzymatic unlabeled **27** are shown in Figures S3 and 3b, respectively. MS analysis of the enzymatic reaction (Figure 3c) demonstrated that unlabeled **27** (163 Da) was formed in excess over singly labeled **27** (165 Da). Since complete exchange would result in a 1:1 mixture, this established that sufficient peroxy oxygen remains in **27** to justify attempting an ^18^O_2_ labeling experiment.

In the event, MS analysis (Figure 3d) of **27** formed with ^18^O_2_ demonstrated the formation of three isotopologues (M, M + 2, and M + 4) corresponding to the incorporation of 0, 1, and 2 atoms of ^18^O (Figure 3d). The M + 4 species is formed by the conversion of ^18^O_2_ labeled peroxide **10** to **27** without exchange of the ketone oxygen. The M + 2 species is formed by the conversion of ^18^O_2_ labeled peroxide **10** to **27** with exchange of one oxygen. This exchange could occur from bound ketoacid intermediates or during hydrazone formation (Figure S2). The high level of the M species was attributed to ^16^O_2_ contamination, to exchange occurring from bound ketoacid intermediates and during hydrazone formation. In addition, repeating the ^18^O_2_ labeling experiment with [4′,5′-^13^C]-PLP^25^ demonstrated that 23% of the nonlabeled hydrazone was derived from a glyoxal contaminant of unknown origin in the reaction buffer (Figure S4).

The observation of ^18^O incorporation from molecular oxygen into the carboxylate of **27** via azadiene **10** and imine **25** provided the first experimental evidence for PLP activation by oxidative dearomatization.

### Model Study to Evaluate the Diels–Alder Reactivity of **29**

**Figure 4:**
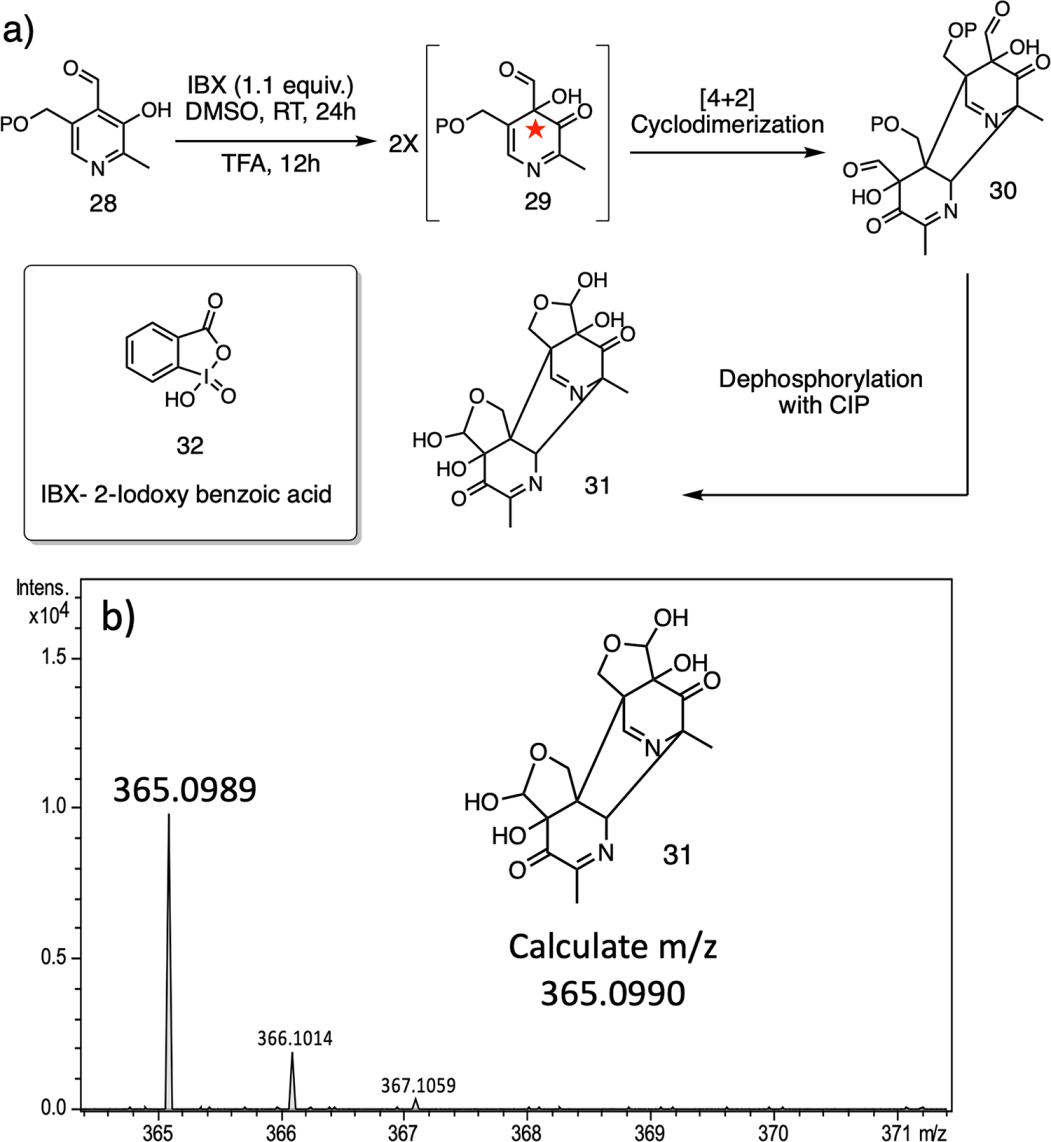
2-Iodoxybenzoic acid (IBX) oxidation of PLP as a model system for the early steps in the THI5p-catalyzed reaction. (a) Oxidative dearomatization of PLP with IBX and dephosphorylation of the resulting product to form **31**. (b) Mass spectrum of **31**.

The formation of the PLP peroxide **10** and its subsequent Diels–Alder reaction to form **11** are new reactions in PLP chemistry. We therefore evaluated the reactivity of **29** to establish the feasibility of these steps (Figure 4a). We selected alcohol **29** rather than peroxide **10** for these studies because of its ease of synthesis. In addition, peroxide **10** and alcohol **29** are likely to show similar Diels–Alder reactivity.

Napthols and 2-alkylphenols can efficiently undergo 2-iodoxybenzoic acid (IBX)-mediated hydroxylative dearomatization to their corresponding orthoquinols.^26−30^ We applied this method to synthesize **29** (Figure 4a).^27^ In the event, **29** proved to be highly reactive, undergoing rapid Diels–Alder dimerization to give **30**, which was characterized by liquid chromatography–mass spectrometry (LC-MS) and NMR after dephosphorylation with CIP and cyclization to give **31** (Figures 4b and S5–S8). Compound **29** did not react with imidazole, presumably because the azadiene (**29**) 5,6 double bond is a better dienophile than the 1,2 double bond of the aromatic imidazole heterocycle.

This model study demonstrates that the proposed oxidative dearomatization of PLP to a Diels–Alder-competent azadiene is chemically feasible. Unfortunately, the high reactivity of **29** precluded its testing as an intermediate in the THI5p-catalyzed reaction.

### Search for Shunt Metabolites

In an enzymatic reaction involving many steps, it is unlikely that the enzyme can bind all of the intermediates sufficiently tightly to fully control the reaction pathway or to prevent the premature release of intermediates. Our mechanistic proposal suggests that intermediates formed before **11** and after **18** may dissociate prematurely from the enzyme (Figure 2). The resulting products would give snapshots of the reaction coordinate. Such snapshots would be of great value in a challenging mechanistic investigation.

**Figure 5:**
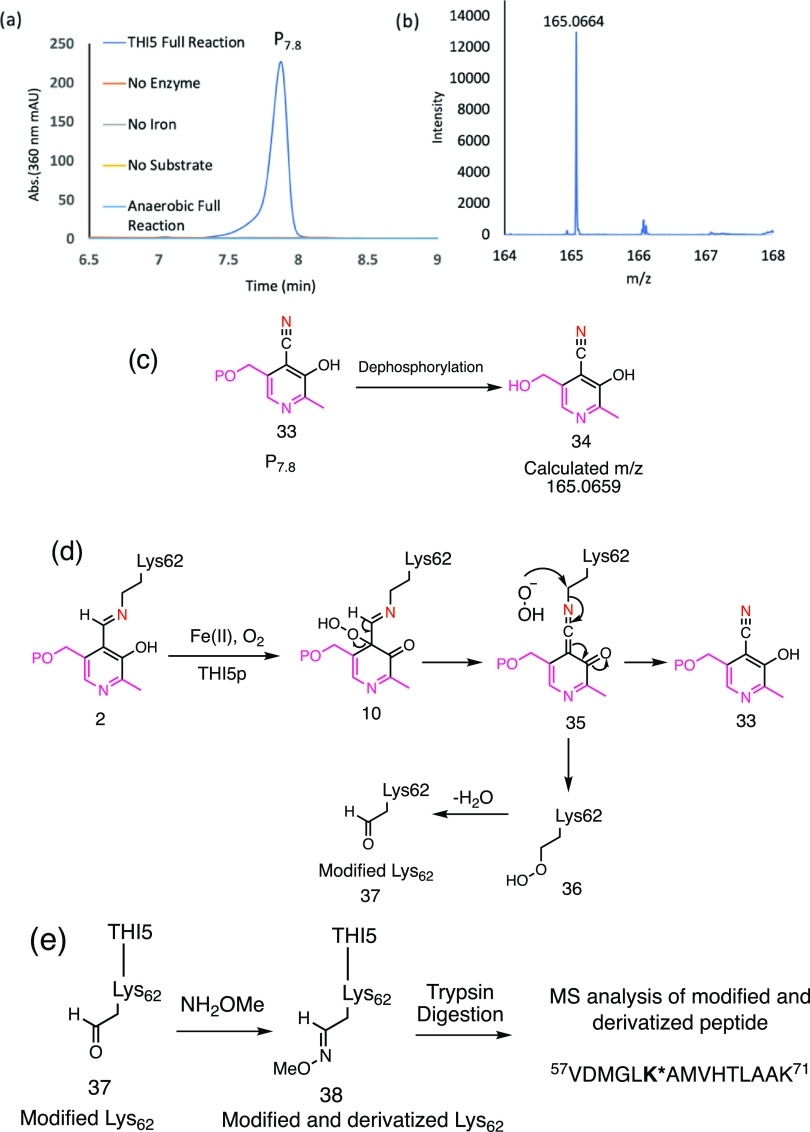
Identification of cyano-PLP (**33**) as a shunt metabolite formed in the native THI5p reaction. (a) HPLC chromatogram showing that P_7.8_ formation requires Fe(II), oxygen, THI5p, and PLP. (b) Mass of dephosphorylated P_7.8_. (c) Structures of P_7.8_ as cyano-PLP (**33**) and its dephosphorylated form as cyano pyridoxal (**34**). (d) Mechanistic proposal for cyano-PLP formation and the resulting lysine modification. (e) Conversion of modified lysine 62 (**37**) to oxime **38** for MS analysis.

High-performance liquid chromatography (HPLC) analysis of the standard THI5p reaction mixture demonstrated the formation of a second reaction product (P_7.8_, Figures 5a and S9). The formation of this product required Fe(II), oxygen, and THI5p (Figure 5a). P_7.8_ had λ_max_ = 360 nm (Figure S9). The new peak was purified by HPLC and analyzed by LC-MS after dephosphorylation with CIP (Figure S10) to give a mass of 165.0664 Da (Figures 5b and S10). P_7.8_ is likely to have a large extinction coefficient because it was not possible to isolate it in sufficient quantities for NMR analysis.

P_7.8_ was prepared from U-^13^C-^15^N-THI5p to identify protein-derived atoms. The resulting dephosphorylated product showed a 1 Da mass increase (Figure S11). This demonstrated that P_7.8_ was not a pyrimidine because incorporation of the His66-derived ^15^N-^13^C-^15^N fragment would result in a 3 Da mass increase. Biosynthesis of P_7.8_ using U-^15^N-THI5p also showed a 1 Da mass increase in dephosphorylated P_7.8_ (Figure S11), demonstrating that P_7.8_ contained one protein-derived nitrogen. The absorbance at 360 nm, the presence of a phosphate, the measured mass, and the transfer of a single nitrogen atom from THI5p to P_7.8_ are all consistent with structure **33** (Figures 5c and S9–S11). This assignment was confirmed by demonstrating that dephosphorylated P_7.8_ (**34**) comigrated with a synthetic sample of **34** (Figures S12–S16).^31^ Since the nitrile nitrogen is the only new atom in **33**, the conversion of PLP to **33** is likely to involve transfer of the amino group of lysine 62.^32^

Our mechanistic proposal for the formation of cyano-PLP is outlined in Figure 5d. Oxidative dearomatization of the PLP imine gives azadiene **10**. In competition with the Diels–Alder reaction, **10** undergoes peroxide elimination to give **35**. Nucleophilic attack of the peroxide on the ε-carbon of Lys62 gives the cyanopyridine **33** and peroxide **36**. Elimination of water from **36** gives aldehyde **37**. Consistent with this proposal, the formation of **33** requires Fe(II), oxygen, and a single nitrogen atom derived from THI5p. The mechanism also predicts the conversion of Lys62 to aldehyde **37**. To test this, the single-turnover inactivated enzyme was derivatized with methoxyamine to convert aldehyde **37** into the corresponding oxime **38** (Figures 5e and S17). Trypsin digestion of the derivatized protein, followed by LC-MS and MS-MS analysis resulted in the identification of the expected modified and derivatized lysine 62 (**38**) in the ^57^VDMGLK*AMVHTLAAK^71^ peptide (Figure S17).^33−35^

### Partial Reconstitution of HMP-P Formation Using THI5p-H66G and Exogenous Imidazole

**Figure 6:**
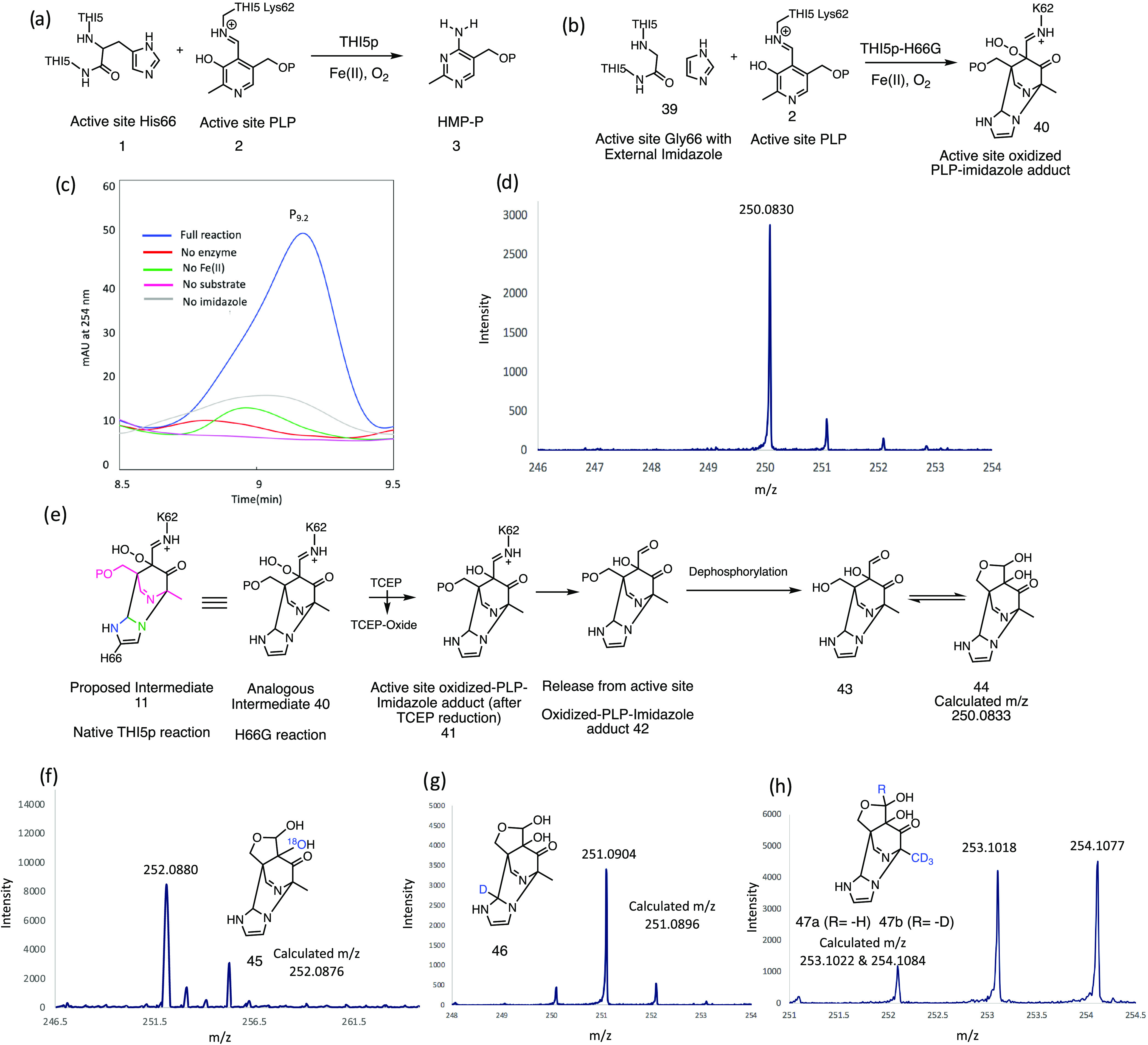
Chemical rescue experiment with THI5p-H66G. (a) Native THI5p reaction. (b) THI5p-H66G reaction showing the structure of the expected product (**40**). (c) HPLC chromatogram of the reaction mixture showing the formation of P_9.2_ in THI5p-H66G full reaction. (d) LC-MS analysis of P_23_ (also see Figure S19). (e) Proposed conversion of intermediate **40** to the observed product **44** (P_23_). (f–h) Mass spectra of the dephosphorylated labeled adducts formed from ^18^O_2_, 2-D-imidazole, and a mixture of 2′-CD_3_-PLP and 4′-formyl-d-2′-CD_3_-PLP, respectively.

The initial steps of our current mechanistic proposal (Figure 2) for the THI5p-catalyzed reaction involve the oxidative dearomatization of PLP to give azadiene **10** followed by a stepwise Diels–Alder reaction with His66 to give **11**. This intermediate is covalently attached to the enzyme via Lys62 and His66. To facilitate the removal of reaction intermediates from the active site, we explored the possibility of carrying out the THI5p reaction using THI5p-H66G and exogenous imidazole (Figure 6a,b). With this system, we hoped that release of intermediates **10** and **11** by imine hydrolysis would be possible, thus facilitating their characterization. Analogous chemical rescue experiments, in which the activity of a mutant enzyme is restored by the addition of a small molecule that compensates for the lost function of the mutated amino acid, have been reported with several other enzymes. In general, a large increase in *K*_m_ and a large reduction in *k*_cat_ are observed in these experiments.^36−40^

The chemical rescue reaction of THI5p-H66G was carried out under standard reaction conditions using varying concentrations of imidazole (10–2000 mM). The reaction was quenched by ultrafiltration and analyzed by HPLC and LC-MS. HPLC analysis showed the formation of a new peak eluting at 9.2 min (P_9.2_, Figures 6c and S18). The extracted ion chromatogram of P_9.2_ demonstrated that its formation required THI5p-H66G, imidazole, Fe(II), and oxygen (Figures 6c and S18). Dephosphorylation of P_9.2_ gave a less polar product (P_23_, Figure S19) with a mass of 250.0830 Da (Figures 6d and S19). These observations are consistent with the THI5p-H66G catalyzed formation of **40**, the expected analogue of the proposed Diels–Alder adduct **11** (Figures 6e and 2). Peroxide reduction with TCEP would form **41**; hydrolysis of the imine of **41** and dephosphorylation (with CIP) would ultimately give **44** with a mass consistent with the observed product (P_23_, Figures 6e, S18, and S19).

It was not possible to prepare P_23_ in sufficient quantities for NMR analysis because it decomposed during HPLC purification/solvent removal. Attempts to stabilize it by derivatization with methoxyamine, pentafluorobenzylhydroxyl amine (PFBHA), and 1,3-diaminopropane were unsuccessful. Attempts to synthesize reference compounds by reacting imidazole with **29** were unsuccessful. We therefore resorted to indirect characterization using isotopically labeled oxygen, imidazole, and PLP.^25,41,42^ When the reaction was run with ^18^O_2_, the expected 2 Da mass increase was observed, when the reaction was run with 2-D-imidazole, the expected 1 Da mass increase was observed and when the reaction was run with a mixture of 2′-CD_3_-PLP and 4′-formyl-d-2′-CD_3_-PLP, the expected 3 and 4 Da mass increases were observed (Figures 6f–h and S20–S24). These labeling experiments demonstrate that P_23_ contains atoms from PLP and from imidazole. The oxygen labeling experiment is consistent with the ^18^O_2_ labeling experiment carried out with native THI5p and supports the hypothesis that PLP is activated by oxidative dearomatization.

### Partial Reconstitution of HMP-P Formation Using THI5p-K62A and Exogenous PLP Oxime

**Figure 7:**
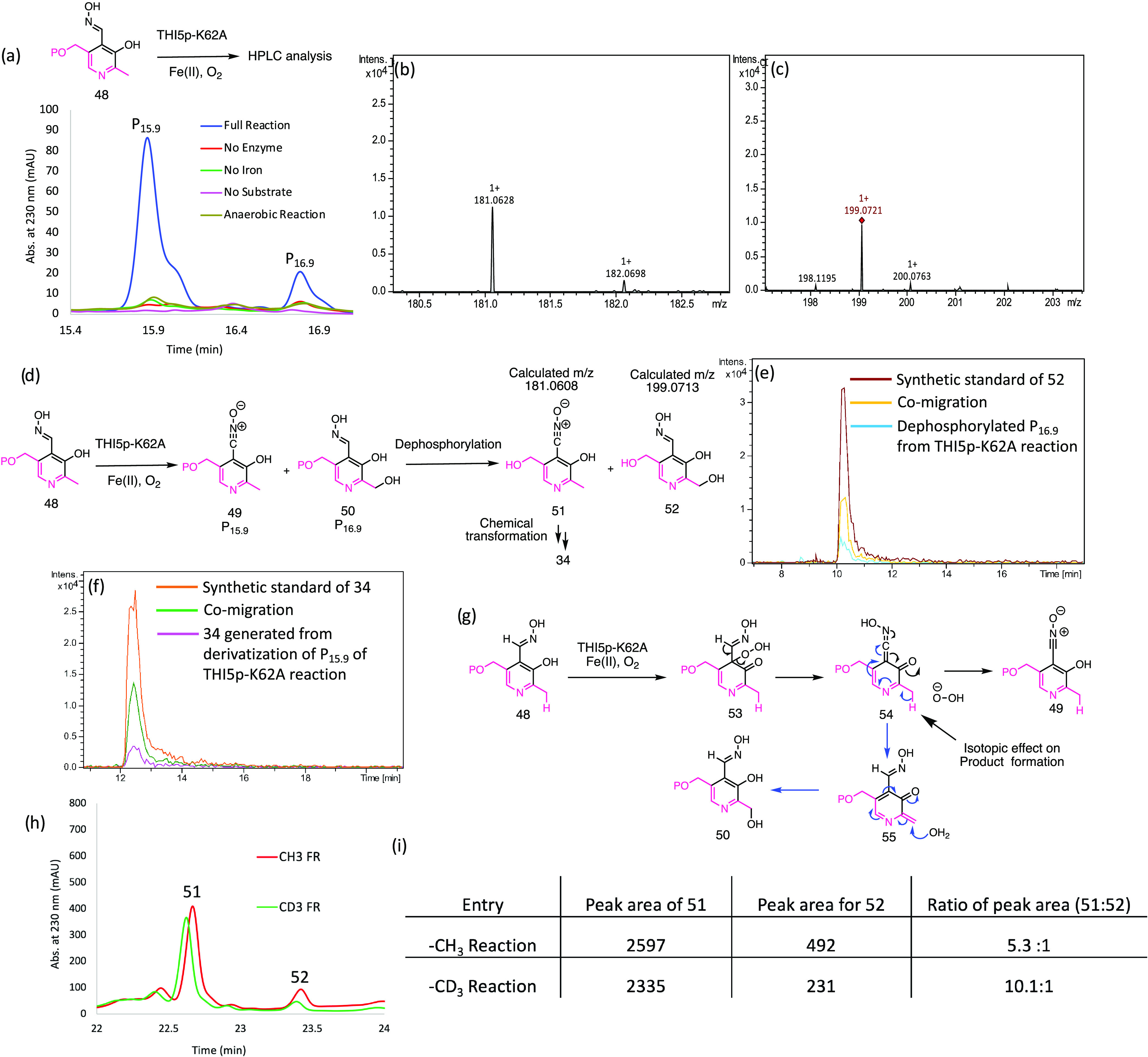
Partial reconstitution of HMP-P formation with THI5p-K62A and PLP oxime **48**. (a) THI5p-K62A catalyzes the conversion of PLP oxime **48** to two new products (P_15.9_ and P_16.9_). HPLC chromatogram shows that the formation of P_15.9_ and P_16.9_ requires Fe(II), oxygen, THI5p-K62A, and PLP oxime **48**. (b, c) Mass of dephosphorylated P_15.9_ and dephosphorylated P_16.9_. (d) Structures of products formed in the THI5p-K62A reaction. (e) LC-MS analysis showing co-migration of dephosphorylated P_16.9_ with a synthetic standard of **52**. (f) LC-MS analysis showing co-migration of derivatized P_15.9_ with a synthetic standard of **34**. (g) Mechanistic proposal for the formation of **49** and **50**. (h) HPLC chromatogram showing the formation of products **51** and **52** in THI5p-K62A reaction (after dephosphorylation) when **48** and 2′-CD_3_-[**48**] were used as substrates. (i) Calculation of the dephosphorylated [**49**]/[**50**] ratio using **48** and 2′-CD_3_-[**48**] as substrates.

The formation of **33** suggested that removal of the bond linking PLP to THI5p might result in the release of intermediates formed prior to the Diels–Alder reaction. We therefore studied the reaction of THI5p-K62A with PLP oxime **48** (Figures 7 and S25–S27).^43^ HPLC analysis of the THI5p-K62A reaction mixture (Figures 7a and S28) demonstrated the formation of two new products that were present only in the complete reaction mixture (P_15.9_ and P_16.9_, Figure 7a). After dephosphorylation, LC-MS analysis gave masses of 181.0628 and 199.0721 Da (Figures 7b,c and S29) consistent with structure **49** for P_15.9_ and structure **50** (or an isomer of **50**) for P_16.9_ (Figure 7d). The structure of dephosphorylated P_16.9_ was confirmed by comigrating with a synthetic standard of **52** (Figures 7e and S30–S32).^44−46^ We were unable to synthesize **51** due to its instability. However, treatment of dephosphorylated P_15.9_, under conditions known to convert nitrile oxides to nitriles, gave a product that comigrated, by HPLC (Figures S33–S36) and LC-MS (Figure 7f), with a synthetic sample of cyano-pyridoxal (**34**) consistent with this structural assignment.^31,47^

Our mechanistic proposal for the formation of **49** and **50** is shown in Figure 7g. Oxidative dearomatization of **48** to give **53** follows the same pathway as the PLP oxidative dearomatization shown in Figure 2 (**7**–**10**). Elimination of hydrogen peroxide gives **54**. Protonation of **54** at the carbonyl group gives **49** (black arrows), while tautomerization gives **55** (blue arrows). Water addition to **55** forms **50**. This mechanism predicts that **49** and **50** are derived from a common intermediate **54** and that the ratio of **49** to **50** should increase if the reaction is carried out using 2′-CD_3_-[**48**] (Figures S37–S40).^25,42^ When this experiment was carried out, the [**49**]:[**50**] ratio (after dephosphorylation) increased from 5.3 with **48** to 10.1 with 2′-CD_3_-[**48**] consistent with this prediction (Figure 7h,i).

## Conclusions

**Figure 8:**
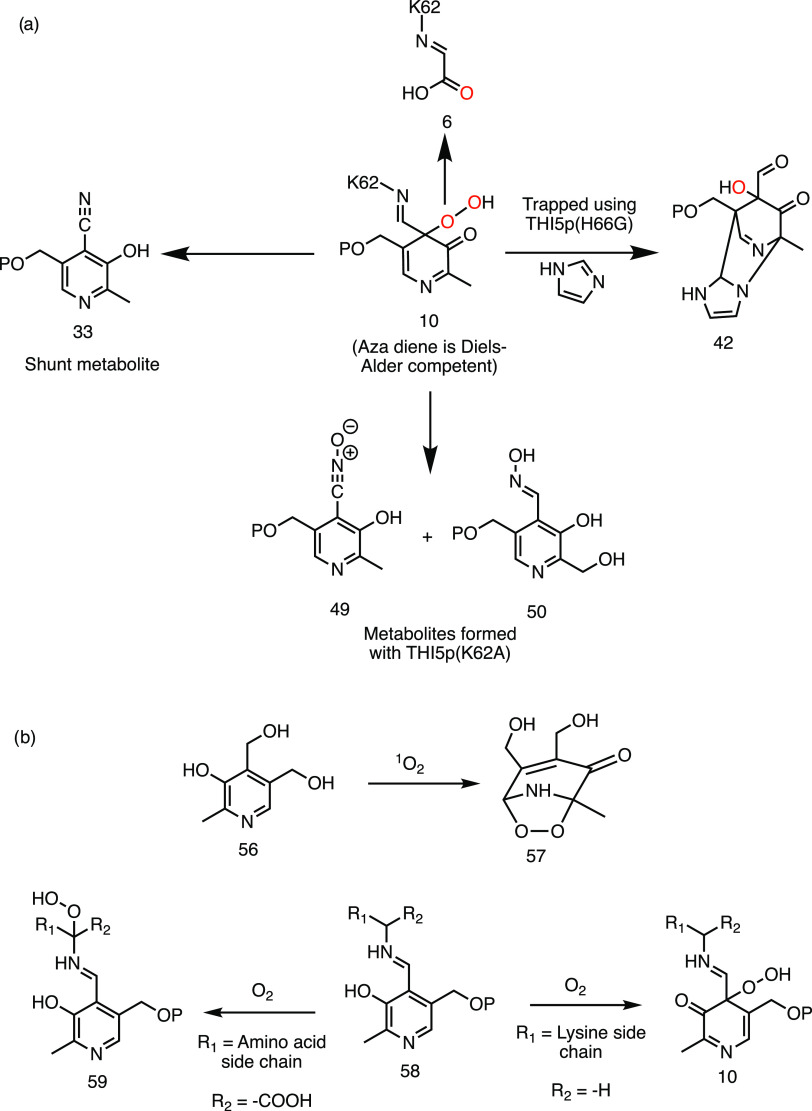
PLP peroxide intermediates and their reactions. (a) Summary of experimental evidence in support of an azadiene peroxide intermediate in the THI5p-catalyzed reaction. (b) Three currently known examples of PLP peroxide intermediates.

In this paper, we continue our investigations on the mechanism of the THI5p-catalyzed formation of the thiamin pyrimidine. Our studies focus on testing the hypothesis that the active-site-bound PLP is activated for a formal Diels–Alder addition of the imidazole of His66 by an oxidative dearomatization to form the azadiene peroxide intermediate (**10**, Figure 2). Our evidence in support of this hypothesis is summarized in Figure 8a and includes the trapping of **10** and the predicted ^18^O_2_ incorporation into **6** and **42**. In addition, the identification of three shunt metabolites (**33**, **49**, and **50**) are all consistent with the intermediacy of azadiene peroxide **10**. Finally, a model study, using a synthesized analogue of **10**, supports the Diels–Alder competence of the PLP-derived azadiene **10**.

Only two examples of PLP peroxides have been previously reported in the biosynthetic literature (Figure 8b). In the first example, PLP and its vitamers are effective quenchers of singlet oxygen and the organic chemistry of the major trapped product **57** has been described.^48^ In the second example, PLP-stabilized carbanions react with molecular oxygen to form peroxides such as **59**, opening up new PLP-mediated redox chemistry.^49^ THI5p system, described in this paper, demonstrates that the azadiene peroxide **10** is a versatile biosynthon participating in new PLP biosynthetic functions. Peroxide chemistry is a new reactivity motif in PLP enzymology, and it is likely that future studies will uncover additional variations of this chemistry.

## Supporting Information Available

Procedures for the synthesis of **31**, **34**, 2-d-imidazole, mixture of 2′-CD_3_-PLP and 4′-formyl-d-2′-CD_3_-PLP, **48**, 2′-CD_3_-[**48**], and **52**; procedures for overexpression and purification of THI5p and its mutants; enzyme reconstitution; tryptic digestion; and MS characterization of small molecules and peptides (PDF)

## Abbreviations

TCEP: tris(2-carboxyethyl) phosphine
PLP: pyridoxal 5′-phosphate
HMP-P: 4-amino-2-methyl-5-hydroxymethylpyrimidine
CIP: calf intestinal alkaline phosphatase
IBX: 2-iodoxybenzoic acid
SIBX: stabilized IBX
EIC: extracted ion chromatogram

## References

[ref1] Begley T. P.; Ealick S. E.; McLafferty F. W. Thiamin Biosynthesis: Still Yielding Fascinating Biological Chemistry. Biochem. Soc. Trans. 2012, 40, 555–560. 10.1042/BST20120084. 22616866 PMC3771315

[ref2] Begley T. P. Cofactor Biosynthesis: An Organic Chemist’s Treasure Trove. Nat. Prod. Rep. 2006, 23, 15–25. 10.1039/b207131m. 16453030

[ref3] Settembre E.; Begley T. P.; Ealick S. E. Structural Biology of Enzymes of the Thiamin Biosynthesis Pathway. Curr. Opin. Struct. Biol. 2003, 13, 739–747. 10.1016/j.sbi.2003.10.006. 14675553

[ref4] Begley T. P.; Ealick S. E. Thiamine: Catalytic Mechanisms in Normal and Disease States; Jordan F.; Patel M. S., Eds.; Marcel Dekker Inc.: New York, 2003; pp 15–28.

[ref5] Spenser I. D.; White R. L. Biosynthesis of Vitamin B1 (Thiamin): An Instance of Biochemical Diversity. Angew. Chem., Int. Ed. 1997, 36, 1032–1046. 10.1002/anie.199710321.

[ref6] Lai R.-Y.; Huang S.; Fenwick M. K.; Hazra A.; Zhang Y.; Rajashankar K.; Philmus B.; Kinsland C.; Sanders J. M.; Ealick S. E.; Begley T. P. Thiamin Pyrimidine Biosynthesis in *Candida albicans*: A Remarkable Reaction between Histidine and Pyridoxal Phosphate. J. Am. Chem. Soc. 2012, 134, 9157–9159. 10.1021/ja302474a. 22568620 PMC3415583

[ref7] Eser B. E.; Zhang X.; Chanani P. K.; Begley T. P.; Ealick S. E. From Suicide Enzyme to Catalyst: The Iron-Dependent Sulfide Transfer in *Methanococcus jannaschii* Thiamin Thiazole Biosynthesis. J. Am. Chem. Soc. 2016, 138, 3639–3642. 10.1021/jacs.6b00445. 26928142 PMC4805478

[ref8] Lai R.-Y.; Mondal A.; Fedoseyenko D.; Begley T. P. Mechanistic Studies on the Single-Turnover Yeast Thiamin Pyrimidine Synthase: Characterization of the Inactive Enzyme. J. Am. Chem. Soc. 2022, 144, 10711–10717. 10.1021/jacs.2c03322. 35675507

[ref9] Chatterjee A.; Abeydeera N. D.; Bale S.; Pai P.-J.; Dorrestein P. C.; Russell D. H.; Ealick S. E.; Begley T. P. *Saccharomyces cerevisiae* Thi4p Is a Suicide Thiamine Thiazole Synthase. Nature 2011, 478, 542–546. 10.1038/nature10503. 22031445 PMC3205460

[ref10] Chatterjee A.; Jurgenson C. T.; Schroeder F. C.; Ealick S. E.; Begley T. P. Thiamin Biosynthesis in Eukaryotes: Characterization of the Enzyme-Bound Product of Thiazole Synthase from *Saccharomyces cerevisiae* and Its Implications in Thiazole Biosynthesis. J. Am. Chem. Soc. 2006, 128, 7158–7159. 10.1021/ja061413o. 16734458 PMC2631426

[ref11] Chatterjee A.; Jurgenson C. T.; Schroeder F. C.; Ealick S. E.; Begley T. P. Biosynthesis of Thiamin Thiazole in Eukaryotes: Conversion of Nad to an Advanced Intermediate. J. Am. Chem. Soc. 2007, 129, 2914–2922. 10.1021/ja067606t. 17309261 PMC2536526

[ref12] Jurgenson C. T.; Chatterjee A.; Begley T. P.; Ealick S. E. Structural Insights into the Function of the Thiamin Biosynthetic Enzyme Thi4 from *Saccharomyces cerevisiae*. Biochemistry 2006, 45, 11061–11070. 10.1021/bi061025z. 16964967

[ref13] Zhang X.; Eser B. E.; Chanani P. K.; Begley T. P.; Ealick S. E. Structural Basis for Iron-Mediated Sulfur Transfer in Archael and Yeast Thiazole Synthases. Biochemistry 2016, 55, 1826–1838. 10.1021/acs.biochem.6b00030. 26919468 PMC4811699

[ref14] Singleton D. A.; Schulmeier B. E.; Hang C.; Thomas A. A.; Leung S.-W.; Merrigan S. R. Isotope Effects and the Distinction between Synchronous, Asynchronous, and Stepwise Diels–Alder Reactions. Tetrahedron 2001, 57, 5149–5160. 10.1016/S0040-4020(01)00354-4.

[ref15] Linder M.; Brinck T. Stepwise Diels–Alder: More Than Just an Oddity? A Computational Mechanistic Study. J. Org. Chem. 2012, 77, 6563–6573. 10.1021/jo301176t. 22780581

[ref16] Linder M.; Johansson A. J.; Brinck T. Mechanistic Insights into the Stepwise Diels–Alder Reaction of 4, 6-Dinitrobenzofuroxan. Org. Lett. 2012, 14, 118–121. 10.1021/ol202913w. 22126093

[ref17] Ishida S.; Tazuya-Murayama K.; Kijima Y.; Yamada K. The Direct Precursor of the Pyrimidine Moiety of Thiamin Is Not Urocanic Acid but Histidine in *Saccharomyces cerevisiae*. J. Nutr. Sci. Vitaminol. 2008, 54, 7–10. 10.3177/jnsv.54.7. 18388401

[ref18] Tazuya K.; Azumi C.; Yamada K.; Kumaoka H. Origin of the N-1, C-2 and C-2′ Atoms of the Pyrimidine Moiety of Thiamin in *Saccharomyces cerevisiae*. Biochem. Mol. Biol. Int. 1994, 33, 769–774. 7981664

[ref19] Tazuya K.; Azumi C.; Yamada K.; Kumaoka H. Pyrimidine Moiety of Thiamin Is Biosynthesized from Pyridoxine and Histidine in *Saccharomyces cerevisiae*. Biochem. Mol. Biol. Int. 1995, 36, 883–888. 8528151

[ref20] Tazuya K.; Morisaki M.; Yamada K.; Kumaoka H. Participation of Histidine in Biosynthesis of the Pyrimidine Moiety of Thiamin in *Saccharomyces cerevisiae*. Biochem. Int. 1988, 16, 955–962. 3048267

[ref21] Tazuya K.; Yamada K.; Kumaoka H. Incorporation of Histidine into the Pyrimidine Moiety of Thiamin in *Saccharomyces cerevisiae*. Biochim. Biophys. Acta, Gen. Subj. 1989, 990, 73–79. 10.1016/S0304-4165(89)80014-5. 2643995

[ref22] Tazuya K.; Yamada K.; Kumaoka H. Pyridoxine Is a Precursor of the Pyrimidine Moiety of Thiamin Is *Saccharomyces cerevisiae*. Biochem. Mol. Biol. Int. 1993, 30, 893–899. 8220238

[ref23] Zeidler J.; Sayer B. G.; Spenser I. D. Biosynthesis of Vitamin B1 in Yeast. Derivation of the Pyrimidine Unit from Pyridoxine and Histidine. Intermediacy of Urocanic Acid. J. Am. Chem. Soc. 2003, 125, 13094–13105. 10.1021/ja030261j. 14570482

[ref24] Liu B.; Wang R.; Xu W.; Zhao G.; Tang L.; Cheng X.; Zhou H. Synthesis and Reaction Mechanism of 3-(4-Methoxyphenylazo) Acrylic Acid. Drug Discoveries Ther. 2009, 3, 93–96. 22495536

[ref25] Chan-Huot M.; Niether C.; Sharif S.; Tolstoy P. M.; Toney M. D.; Limbach H.- H. Nmr Studies of the Protonation States of Pyridoxal-5′-Phosphate in Water. J. Mol. Struct. 2010, 976, 282–289. 10.1016/j.molstruc.2010.03.032.

[ref26] Harned A. M. Asymmetric Oxidative Dearomatizations Promoted by Hypervalent Iodine (Iii) Reagents: An Opportunity for Rational Catalyst Design?. Tetrahedron Lett. 2014, 55, 4681–4689. 10.1016/j.tetlet.2014.06.051. 25147412 PMC4135387

[ref27] Lebrasseur N.; Gagnepain J.; Ozanne-Beaudenon A.; Léger J.-M.; Quideau S. Efficient Access to Orthoquinols and Their [4+2] Cyclodimers Via Sibx-Mediated Hydroxylative Phenol Dearomatization. J. Org. Chem. 2007, 72, 6280–6283. 10.1021/jo0708893. 17628111

[ref28] Ozanne A.; Pouységu L.; Depernet D.; Francois B.; Quideau S. A Stabilized Formulation of IBX (sIBX) for Safe Oxidation Reactions Including a New Oxidative Demethylation of Phenolic Methyl Aryl Ethers. Org. Lett. 2003, 5, 2903–2906. 10.1021/ol0349965. 12889904

[ref29] Pouységu L.; Sylla T.; Garnier T.; Rojas L. B.; Charris J.; Deffieux D.; Quideau S. Hypervalent Iodine-Mediated Oxygenative Phenol Dearomatization Reactions. Tetrahedron 2010, 66, 5908–5917. 10.1016/j.tet.2010.05.078.

[ref30] Quideau S.; Pouységu L. Synthetic Uses of Orthoquinone Monoketals and Their Orthoquinol Variants. A Review. Org. Prep. Proced. Int. 1999, 31, 617–680. 10.1080/00304949909355348.

[ref31] Quinn D. J.; Haun G. J.; Moura-Letts G. Direct Synthesis of Nitriles from Aldehydes with Hydroxylamine-O-Sulfonic Acid in Acidic Water. Tetrahedron Lett. 2016, 57, 3844–3847. 10.1016/j.tetlet.2016.07.047.

[ref32] Sakaki K.; Ohishi K.; Shimizu T.; Kobayashi I.; Mori N.; Matsuda K.; Tomita T.; Watanabe H.; Tanaka K.; Kuzuyama T.; Nishiyama M. A Suicide Enzyme Catalyzes Multiple Reactions for Biotin Biosynthesis in Cyanobacteria. Nat. Chem. Biol. 2020, 16, 415–422. 10.1038/s41589-019-0461-9. 32042199

[ref33] Gundry R. L.; White M. Y.; Murray C. I.; Kane L. A.; Fu Q.; Stanley B. A.; Van Eyk J. E. Preparation of Proteins and Peptides for Mass Spectrometry Analysis in a Bottom-up Proteomics Workflow. Curr. Protoc. Mol. Biol. 2010, 90, 10.25. 11–10.25. 23. 10.1002/0471142727.mb1025s88. PMC2905857 19816929

[ref34] Biemann K. Contributions of Mass Spectrometry to Peptide and Protein Structure. Biomed. Environ. Mass Spectrom. 1988, 16, 99–111. 10.1002/bms.1200160119. 3072035

[ref35] ROEPSTORFE P. Proposal for a Common Nomenclature for Sequence Ions in Mass Spectra of Peptides. Biomed. Mass Spectrom. 1984, 11, 601–605. 10.1002/bms.1200111109. 6525415

[ref36] Peracchi A. How (and Why) to Revive a Dead Enzyme: The Power of Chemical Rescue. Curr. Chem. Biol. 2008, 2, 32–49. 10.2174/2212796810802010032.

[ref37] Drueckes P.; Schinzel R. Activation of E350a Mutant Maltodextrin Phosphorylase by Exogenously Added Acetate. Protein Eng., Des. Sel. 1996, 9, 701–705. 10.1093/protein/9.8.701. 8875647

[ref38] He Z.; Toney M. D. Direct Detection and Kinetic Analysis of Covalent Intermediate Formation in the 4-Amino-4-Deoxychorismate Synthase Catalyzed Reaction. Biochemistry 2006, 45, 5019–5028. 10.1021/bi052216p. 16605270

[ref39] Moracci M.; Trincone A.; Perugino G.; Ciaramella M.; Rossi M. Restoration of the Activity of Active-Site Mutants of the Hyperthermophilic b-Glycosidase from Sulfolobus Solfataricus: Dependence of the Mechanism on the Action of External Nucleophiles. Biochemistry 1998, 37, 17262–17270. 10.1021/bi981855f. 9860840

[ref40] Williams D. M.; Wang D.; Cole P. A. Chemical Rescue of a Mutant Protein- Tyrosine Kinase. J. Biol. Chem. 2000, 275, 38127–38130. 10.1074/jbc.C000606200. 11006267

[ref41] Proniewicz L. M.; Bruha A.; Nakamoto K.; Kyuno E.; Kincaid J. R. Resonance Raman Spectra of Dioxygen Adducts of Cobalt Porphyrin-Imidazole Complexes. Remarkable Spectroscopic Consequences of Hydrogen Bonding of the Coordinated Imidazole and the Lack of an Effect on the Cobalt-Oxygen Linkage. J. Am. Chem. Soc. 1989, 111, 7050–7056. 10.1021/ja00200a024.

[ref42] Coburn S. P.; Lin C.; Schaltenbrand W.; Mahuren J. Synthesis of Deuterated Vitamin B6 Compounds. J. Labelled Compd. Radiopharm. 1982, 19, 703–716. 10.1002/jlcr.2580190510.

[ref43] Heyl D.; Luz E.; Harris S. A.; Folkers K. Phosphates of the Vitamin B6 Group. I. The Structure of Codecarboxylase. J. Am. Chem. Soc. 1951, 73, 3430–3433. 10.1021/ja01151a126.

[ref44] Pocker A. Synthesis of 2-nor-2-Formylpyridoxal 5′-Phosphate, a Bifunctional Reagent Specific for the Cofactor Site in Proteins. J. Org. Chem. 1973, 38, 4295–4299. 10.1021/jo00964a019. 4791802

[ref45] Dale T. J.; Sather A. C.; Rebek J. Jr Synthesis of Novel Aryl-1, 2-Oxazoles from Ortho-Hydroxyaryloximes. Tetrahedron Lett. 2009, 50, 6173–6175. 10.1016/j.tetlet.2009.08.086.

[ref46] Weeks K. L.; Rutkowski K. R.; Loyola A. A. M.; Boyce G. R. Utilization of Pyridoxal Acetal Salts as Water-Triggered, Slow-Release Pro-Fragrances. New J. Chem. 2018, 42, 15538–15540. 10.1039/C8NJ03229G.

[ref47] Aitken R. A.; Raut S. V. A Convenient Mild Two-Step Conversion of Imines to Secondary and Tertiary Amides. Synlett 1991, 1991, 189–190. 10.1055/s-1991-20674.

[ref48] Samuel D.; Norrell K.; Hilmey D. G. Novel Ring Chemistry of Vitamin B6 with Singlet Oxygen and an Activated Ene: Isolated Products and Identified Intermediates Suggesting an Operable [3+ 2] Cycloaddition. Org. Biomol. Chem. 2012, 10, 7278–7281. 10.1039/c2ob26067k. 22893184

[ref49] Hoffarth E. R.; Rothchild K. W.; Ryan K. S. Emergence of Oxygen-and Pyridoxal Phosphate-Dependent Reactions. FEBS J. 2020, 287, 1403–1428. 10.1111/febs.15277. 32142210

