# Supplementary material for: Oxidative Dearomatization of PLP in Thiamin Pyrimidine Biosynthesis in Candida albicans
Source: J Am Chem Soc. 2023 Feb 20;145(8):4421–30. doi: 10.1021/jacs.2c08560 (PMC10848271; doi:10.1021/jacs.2c08560)
Supplement: Supplementary file 1 — ja2c08560_si_001.pdf [file ja2c08560_si_001.pdf]

Oxidative Dearomatization of PLP in Thiamin Pyrimidine Biosynthesis in Candida albicans

Anushree Mondal, Rung-Yi Lai[§], Dmytro Fedoseyenko, Nitai Giri[¶], Tadhg P. Begley*

Department of Chemistry, Texas A&M University, College Station, Texas 77843, United States

[§]Rung-Yi Lai's current address - School of Chemistry, Institute of Science, Suranaree University of Technology, 111 University Avenue, Muang, Nakhon Ratchasima, Thailand 30000.

[¶]Nitai Giri's current address - Central Institute of Petrochemical Engineering and Technology, Raipur-493221, Chhattisgarh, India.

(*To whom correspondence should be addressed)

Supporting Information

Table of Contents:

Abbreviations	3
Materials	3
Overexpression and purification of THI5p and its mutants	3
Overexpression and purification of ^{15}N-THI5p and ^{13}C-^{15}N THI5p	4
Overexpression and purification of PdxK	4
HPLC and LC-MS conditions	4-6
Oxygen labelling studies on PLP by -product **6**	6-8
Synthesis of **31**	9-10
Enzymatic reaction condition for $P_{7.8}$ formation	11-12
Synthesis of **34**	13-14
Quantitation of cyano PLP (**33**) formed in THI5p reaction	14-15
Condition for trypsin digestion of single-turnover inactivated THI5p reaction	15-16
Enzymatic reaction condition chemical rescue experiment with THI5p-H66G	17-19
Synthesis of labelled imidazole and labelled PLP analogs	20-21
Synthesis of **48**	21-23
Conditions for the THI5p-K62A-catalyzed reaction	23-24
Synthesis of **52**	24-25
Characterization of $P_{15.9}$ from the THI5p-K62A catalyzed reaction	26-28
Synthesis of 2'- CD_3-[**48]**	28-31
References	32-33

Abbreviations

IPTG, isopropyl-β-D-1-thiogalactopyranoside; DTT, DL-dithiothreitol; TCEP, tris(2-carboxyethyl) phosphine; SDS-PAGE, sodium dodecyl sulfate polyacrylamide gel electrophoresis; PLP, pyridoxal 5′ -phosphate; HMP-P, 4-amino-2-methyl-5-hydroxymethylpyrimidine; IBX, 2-iodoxy benzoic acid; SIBX, stabilized IBX; CIP, calf intestinal alkaline phosphatase; EIC, extracted ion chromatogram.

Materials

All chemicals were purchased form Sigma-Aldrich (or, now Millipore Sigma) unless mentioned otherwise. M9 medium was purchased from VWR life sciences. IPTG and Kanamycin sulfate were obtained from Lab Scientific Inc and Teknova respectively. Histrap columns were obtained from GE Healthcare. 10DG desalting columns were obtained from Bio-Rad. Amicon Ultra centrifugal filter devices (10,000 MWCO) were obtained from Millipore. 2.5 L baffled ultra-yield flasks for protein overexpression were obtained from Thomson Instrument Company. Centrifugal filters for protein filtering were obtained from VWR. Calf intestinal phosphatase (CIP) was obtained from New England Biolabs. Sequencing grade trypsin was obtained from Promega. Guanidine hydrochloride was obtained from Amresco. Methoxy amine hydrochloride and DTT was obtained from TCI America. Ferrous ammonium sulfate hexahydrate was obtained from Alfa Aesar. TCEP and SIBX was obtained from AK Scientific. Potassium phosphate dibasic was obtained from Oakwood Chemicals. Deuterium oxide, ^{15}N-ammonium chloride and DMSO-d_6 were obtained from Cambridge Isotopic Laboratory. Suplecosil LC-18 HPLC column (5 μm particle size, L x I.D. 25 cm x 10 mm) and Discovery C18 HPLC column (5 μm particle size, L x I.D. 25 cm x 10 mm) were purchased from Sigma-Aldrich. Infinity Lab Poroshell 120 EC-C18 column (2.7 μm particle size, 3 x 100 mm) and ZORBAX Eclipse XDB-C18 HPLC column (4.6 X 150 mm, 5 μm particle size) were purchased from Agilent Technologies. HPLC grade solvents were purchased from Fisher Chemicals and LC-MS grade solvents were purchased from Avantor.

Overexpression and Purification of THI5p and its mutants

E.coli BL21(DE3) cells containing the THI5p gene in the pET28b vector were grown in minimal media (11.3 g M9 salts, 13.3 mL 50% glucose, 2.7 mL 1 M $MgSO_4$, 100 μL 1 M $CaCl_2$ diluted to 1 L) containing kanamycin (40 mg/mL) with shaking at 37 °C until the OD_{600} reached 0.6 (without supplementation of any externally added Fe-salt to minimize the production of inactive protein during overexpression and purification process). Protein overexpression was then induced with IPTG (final concentration of 500 μM), and cell growth was continued at 15 °C for 16 h. The cells were harvested by centrifugation, and the cell pellets from 1 L of culture were resuspended in 20 mL of lysis buffer (10 mM imidazole, 300 mM NaCl, 50 mM NaH_2PO_4, 5 mM DTT, pH 8.0) and lysed by sonication (Heat System Ultrasonics Model W-385 sonicator, 1.5 s cycle, 60% duty). The resulting cell lysate was clarified by centrifugation and the THI5p protein was purified on a Ni-NTA column following the manufacturer's instructions. After elution, the protein was desalted under anaerobic conditions using an Econo-Pac 10DG column (BioRad) pre-equilibrated with 100 mM Tris-HCl buffer, 5 mM DTT, 30% glycerol, pH 7.5. The purified protein was stored in aliquots in liquid nitrogen. All mutants used in this study (H66G and K62A) were also purified using this protocol.

^{15}N-THI5p overexpression and purification

The overexpression and purification procedures were the same as described above for THI5p except $^{14}NH_4Cl$ was replaced with $^{15}NH_4Cl$ in the M9 salts.

^{13}C-^{15}N-THI5p overexpression and purification

The overexpression and purification process for ^{13}C-^{15}N-THI5p was the same as described above except glucose and ammonium chloride were replaced with ^{13}C-glucose and ^{15}N-ammonium chloride.

Overexpression and purification of Pyridoxal Kinase (PdxK)

E.coli BL21(DE3) cells containing the PdxK gene in the pET18b vector were grown in LB media containing ampicilin (100 mg/mL) with shaking at 37 °C until the OD_{600} reached 0.6. Protein overexpression was then induced with IPTG (final concentration of 500 μM), and cell growth was continued at 15 °C for 16 h. The cells were harvested by centrifugation, and the cell pellets from 1 L of culture were resuspended in 20 mL of lysis buffer (10 mM imidazole, 300 mM NaCl, 50 mM NaH_2PO_4, 5 mM DTT, pH 8.0) and lysed by sonication (Heat System Ultrasonics Model W-385 sonicator, 1.5 s cycle, 60% duty). The resulting cell lysate was clarified by centrifugation and the PdxK protein was purified on a Ni-NTA column following the manufacturer's instructions. After elution, the protein was desalted using an Econo-Pac 10DG column (BioRad) pre-equilibrated with 100 mM Tris-HCl buffer, 5 mM DTT, 30% glycerol, pH 7.5. The purified protein was stored in aliquots in liquid nitrogen.

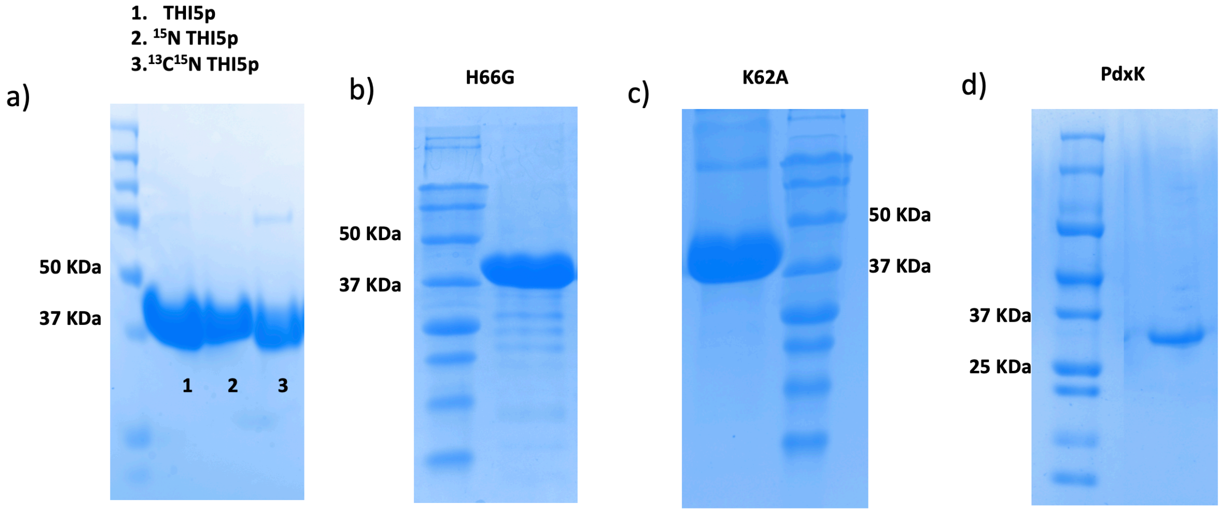

Figure S1. SDS-PAGE of purified proteins. a) THI5p and its isotopologues. (b) THI5p-H66G c) THI5p-K62A d) PdxK. Molecular weight of THI5p is 41 kDa and PdxK is 32 kDa.

HPLC and LC-MS Conditions

HPLC Condition 1

An Agilent 1260 HPLC equipped with a quaternary pump was used. The system included a diode array UV-VIS detector. The following linear gradient, at a flow rate of 2 mL/min, on a Supelcosil LC-18 column (250 mm X 10 mm, 5 μm ID) was used: solvent A is water, solvent B is

100 mM KH_2PO_4, pH 6.6, solvent C is methanol; 0 min – 100%B, 5 min – 100%B, 10 min – 25%A 60%B 15%C, 13 min - 25%A 60%B 15%C, 17 min - 30%A 10%B 60%C, 21 min - 100%B, 32 min - 100%B.

HPLC Condition 2

An Agilent 1260 HPLC equipped with a quaternary pump was used. The system included a diode array UV-VIS detector. The following linear gradient, at a flow rate of 2 mL/min, on a Supelcosil LC-18 column (250 mm X 10 mm, 5 µm ID) was used: solvent A is water, solvent B is 10 mM Ammonium acetate, pH 6.6, solvent C is methanol; 0 min – 100%B, 5 min – 100%B, 10 min – 25%A 60%B 15%C, 13 min - 25%A 60%B 15%C, 17 min - 30%A 10%B 60%C, 21 min - 100%B, 32 min - 100%B.

HPLC Condition 3

An Agilent 1260 HPLC equipped with a quaternary pump was used. The system included a diode array UV-VIS detector. The following linear gradient, at a flow rate of 2 mL/min, on a Discovery LC-18 column (250 mm X 10 mm, 5 µm ID) was used: solvent A is water, solvent B is 100 mM KH_2PO_4, pH 6.6, solvent C is methanol; 0 min – 100%B, 5 min – 100%B, 10 min – 25%A 60%B 15%C, 13 min - 25%A 60%B 15%C, 17 min - 30%A 10%B 60%C, 21 min - 100%B, 32 min - 100%B.

HPLC Condition 4

An Agilent 1260 HPLC equipped with a quaternary pump was used. The system included a diode array UV-VIS detector. The following linear gradient, at a flow rate of 2 mL/min, on a Discovery LC-18 column (250 mm X 10 mm, 5 µm ID) was used: solvent A is water, solvent B is 10 mM ammonium acetate, pH 6.6, solvent C is methanol; 0 min – 100%B, 5 min – 100%B, 10 min – 25%A 60%B 15%C, 13 min - 25%A 60%B 15%C, 17 min - 30%A 10%B 60%C, 21 min - 100%B, 32 min - 100%B.

HPLC Condition 5

An Agilent 1260 HPLC equipped with a quaternary pump was used. The system included a diode array UV-VIS detector. The following linear gradient, at a flow rate of 1 mL/min, on a Zorbax Eclipse XBD-LC-18 column (4.6 X 150 mm, 5 µm particle size) was used: solvent A is 5 mM ammonium acetate, pH 6.6, solvent B is 75% methanol; 0 min – 100% A, 2 min – 100%A, 12 min – 30% A 70% B, 17 min – 30% A 70% B, 18 min -100% A, 20 min - 100% A.

HPLC Condition 6

An Agilent 1260 HPLC equipped with a quaternary pump was used. The system included a diode array UV-VIS detector. The following linear gradient, at a flow rate of 1 mL/min, on a Zorbax Eclipse XBD-LC-18 column (4.6 X 150 mm, 5 µm particle size) was used: solvent A is water, solvent B is 100 mM KH_2PO_4, pH 6.6, solvent C is methanol; 0 min – 100%B, 5 min – 100%B, 10 min – 25%A 60%B 15%C, 13 min - 25%A 60%B 15%C, 17 min - 30%A 10%B 60%C, 21 min - 100%B, 32 min - 100%B.

LCMS Condition 1

LC-ESI-ToF-MS was performed using an Agilent 1260 HPLC system equipped with a binary pump and 1200 series diode array detector followed by a MicroToF-Q II mass spectrometer (Bruker Daltonics) using an ESI source either in negative or positive mode. The analysis was performed on an InfinityLabPoroshell 120 LC column (2.7 μm, 3X100 mm). General injection volumes were in the range of 80-90 μL. The data was processed using DataAnalysis 4.0 SP 1 (Bruker Daltonics).LC conditions, A – 5mM ammonium acetate buffer, pH 6.6, B – 75% methanol and 25% water, LC method , 0 min – 100% A, 2 min – 100%A, 12 min – 30% A 70% B, 17 min – 30% A 70% B, 18 min -100% A, 20 min - 100% A.

LC-MS Condition 2

The following liner gradient, at a flow rate of 0.4 μL/min on a Acclaim Prepmap 100 C18 LC column (150mm length and 3 μm particle size) was used : Solvent A is water with 0.1% Formic acid and solvent B is Acetonitrile with 0.1% Formic acid ; 0 min : 98% A 2% B ; 5 min: 98% A 2% B ; 37 min: 55% A 45% B ; 40 min: 10% A 90% B ; 46 min: 10% A 90% B 47 min: 98% A 2% B ; 60 min: 98% A 2% B. The HPLC was couple with Orbitrap Fusion Tribird for MS analysis in the positive mode.

Oxygen labelling studies on PLP-by product 6

Reconstitution of THI5p activity

The THI5p reaction was performed in 100 mM HEPES buffer with 1mM TCEP, pH 7.5 containing THI5p(240 μM), $Fe(NH_4)_2(SO_4)_2$ (120 μM) and PLP (480 μM). The enzyme was anaerobically preincubated with the iron for 30 mins in an ice bath. Then PLP was added followed by another anaerobic incubation at room temperature for 30 mins. Finally the mixture was aerobically incubated at room temperature for 3h. The reaction was quenched by filtering with a 10 kDa cut-off filter.

THI5p reaction in 50% H_2O^{18} buffer

THI5p reaction was carried out as per the protocol described above except the HEPES buffer (H_2O) was replaced with H_2O/H_2O^{18} (1:1) HEPES buffer.

THI5p reaction with $^{18}O_2$

THI5p reaction was carried out as described above except during the aerobic incubation part the reaction mixture was exposed to $^{18}O_2$ rather than $^{16}O_2$.

THI5p reaction with $^{18}O_2$ and [4',5'-^{13}C]-PLP

THI5p reaction was carried out with [4',5'-^{13}C]-PLP as the substrate as described above except $^{16}O_2$ was replaced with $^{18}O_2$. The synthesis of [4',5'-^{13}C]-PLP was carried out as previously reported.[1, 2]

Trapping of the PLP-derived by-product with phenylhydrazine

After the THI5p reaction is quenched by ultrafiltration, 10 μL of phenylhydrazine stock solution (5 μL in 1 mL of water, 50 mM) was added to the filtrate. The mixture was incubated at 37 °C for 2.5 h followed by LC-MS analysis (LC-MS condition 1).[2, 3]

Synthesis of 27

The synthesis of **27** was carried out as previously reported.[2, 3]

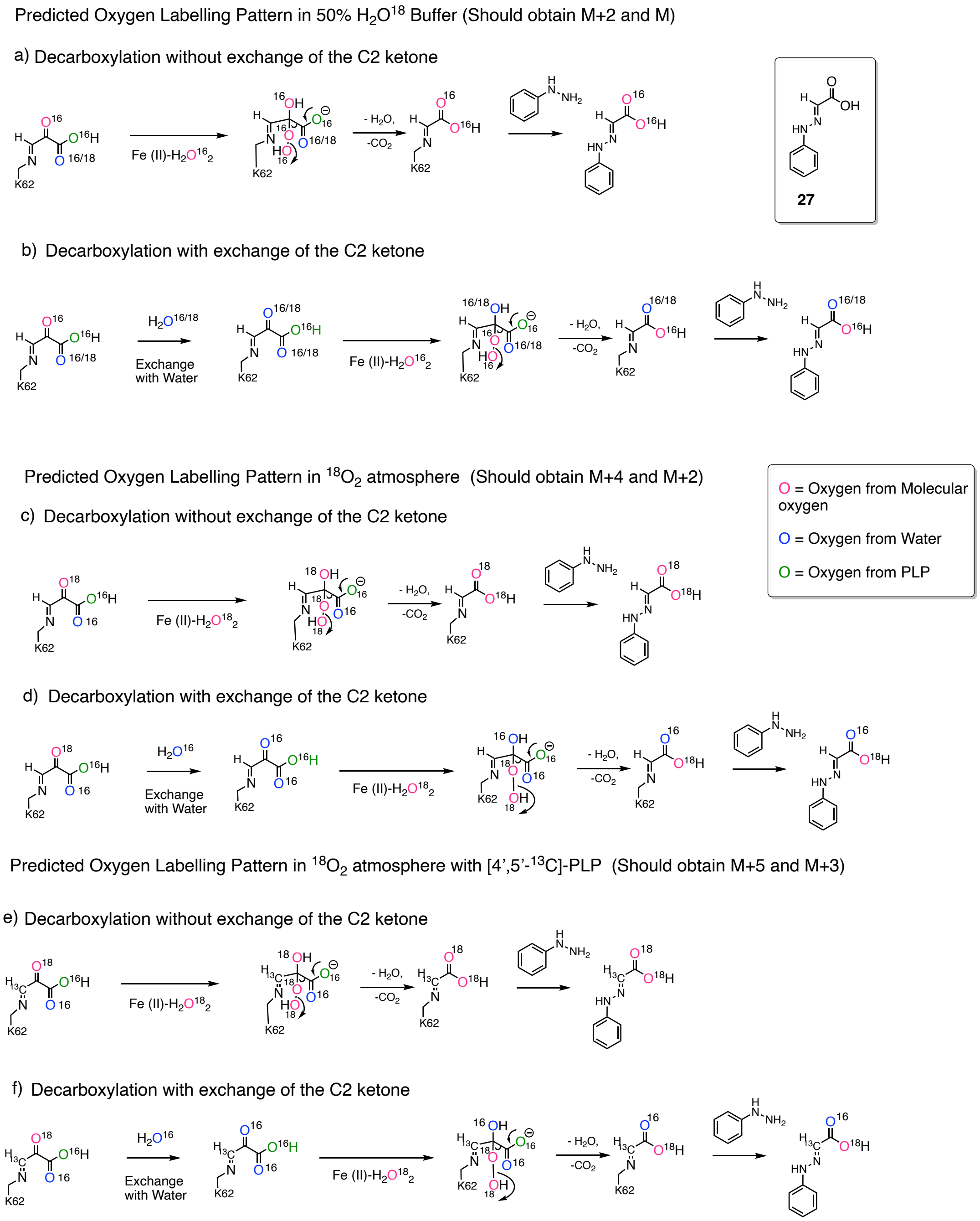

Figure S2. Predicted oxygen labelling pattern in PLP by-product **27** (general structure without any labelling is shown in the inset). The oxygen labelling pattern is shown in two ways considering the possibility of solvent exchange. a) and b) Oxygen labelling pattern when the reaction was run in 50% H_2O^{18}. c) and d) Oxygen labelling pattern when $^{18}O_2$ is used. e) and f) Oxygen labelling pattern with $^{18}O_2$ and [4',5'-^{13}C]-PLP is used with $^{18}O_2$.

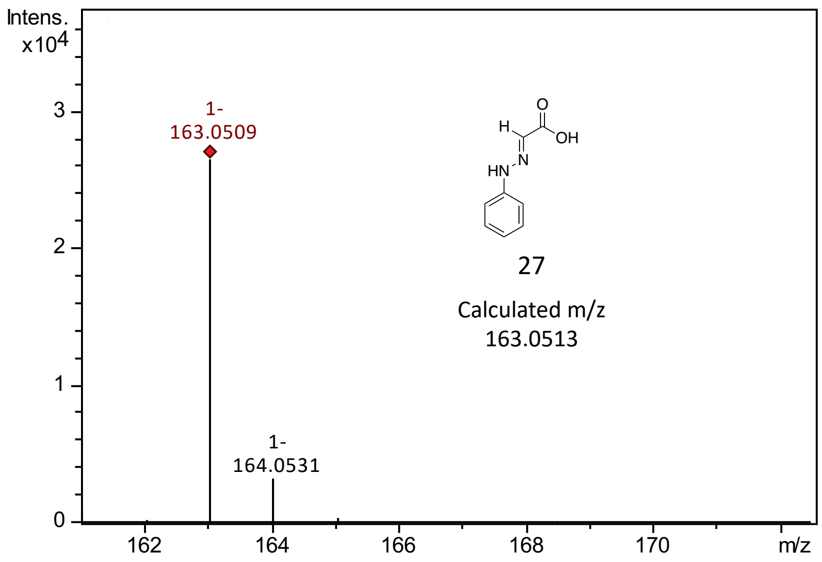

Figure S3. MS spectrum of synthetically prepared **27**.

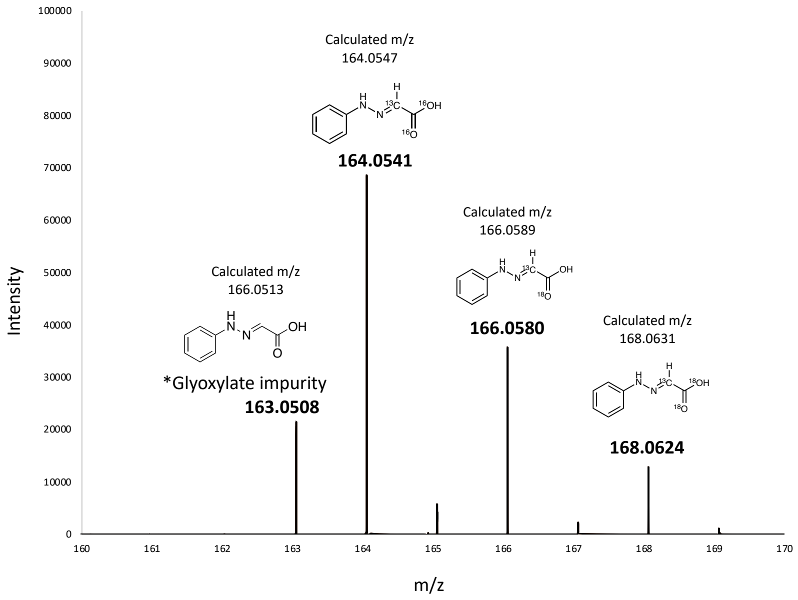

Figure S4. MS spectrum of **27** in the $^{18}O_2$ reaction with [4',5'-^{13}C]-PLP showing M+3 (166.0580 Da) and M+5 (168.0624 Da) peaks. This experiment indicates that oxygen from azadiene **10** is retained in the glyoxylate hydrazone. The M peak (163.0513 Da) is due to a glyoxylate impurity in the buffer and the M+1 peak is due to a $^{16}O_2$ contamination.

Synthesis of 31

To a solution of starting pyridoxal phosphate (10 mmol) in DMSO (25 mL) was added SIBX (11 mmol) as a solid in one portion. The resulting suspension was stirred at room temperature for 24 hours, after which time TFA (780 µL) was added, and the mixture was further stirred for 12 hours. The reaction mixture was then diluted with water (50 mL) and neutralized with a saturated solution of aq. Na_2CO_3.[4] The reaction mixture was CIP treated before HPLC analysis and purification (HPLC condition 5) and LC-MS (condition 1) analysis. Mass in negative mode 365.0989 Da. ^{1}H NMR (400 MHz, DMSO-d_6): δ 8.005 (s, 1H), 5.854 (s, 1H), 5.744 (s, 1H), 4.001-3.995 (d, 1H, J=2.4 Hz), 3.980-3.973 (d, 1H, J=2.8 Hz), 3.729-3.724 (d, 1H, J=2 Hz), 3.707-3.703 (d, 1H, J=1.6 Hz), 3.526 (s, 3H), 2.386 (s, 3H), 1.246 (s, 1H). ^{13}C NMR (125 MHz, D_2O): δ 164.553, 159.419, 148.402, 139.019, 99.987, 99.358, 97.270, 96.693, 73.446, 73.135, 72.090, 62.757, 62.535, 37.750, 15.261.

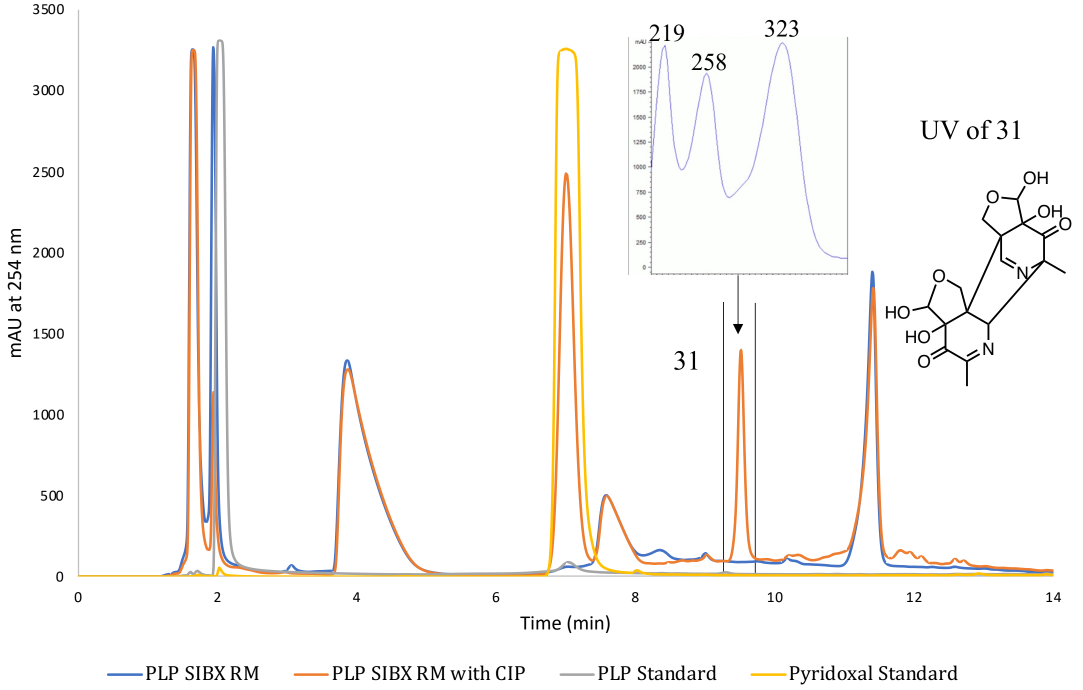

Figure S5. HPLC Chromatogram for collection and purification of **31**. Inset: UV of **31**.

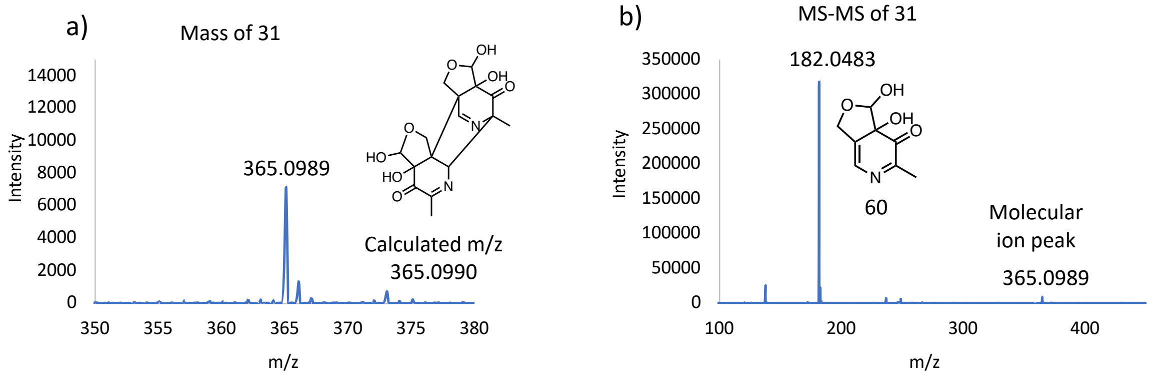

Figure S6. LC-MS analysis of **31**. a) Mass of purified **31**. b) MS/MS fragmentation spectra of **31** showing the mass of its monomer **60** as the predominant fragment peak.

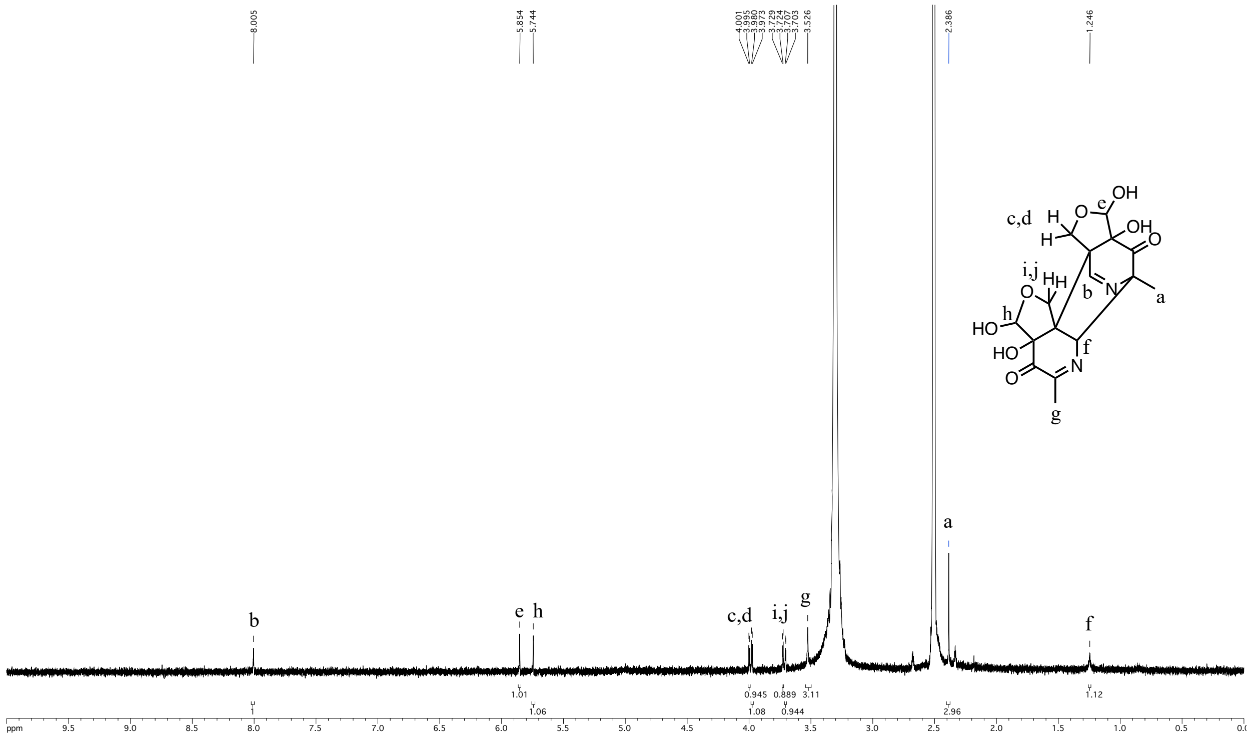

Figure S7. ^{1}H NMR Spectrum (400 MHz, DMSO-d_6) for **31**.

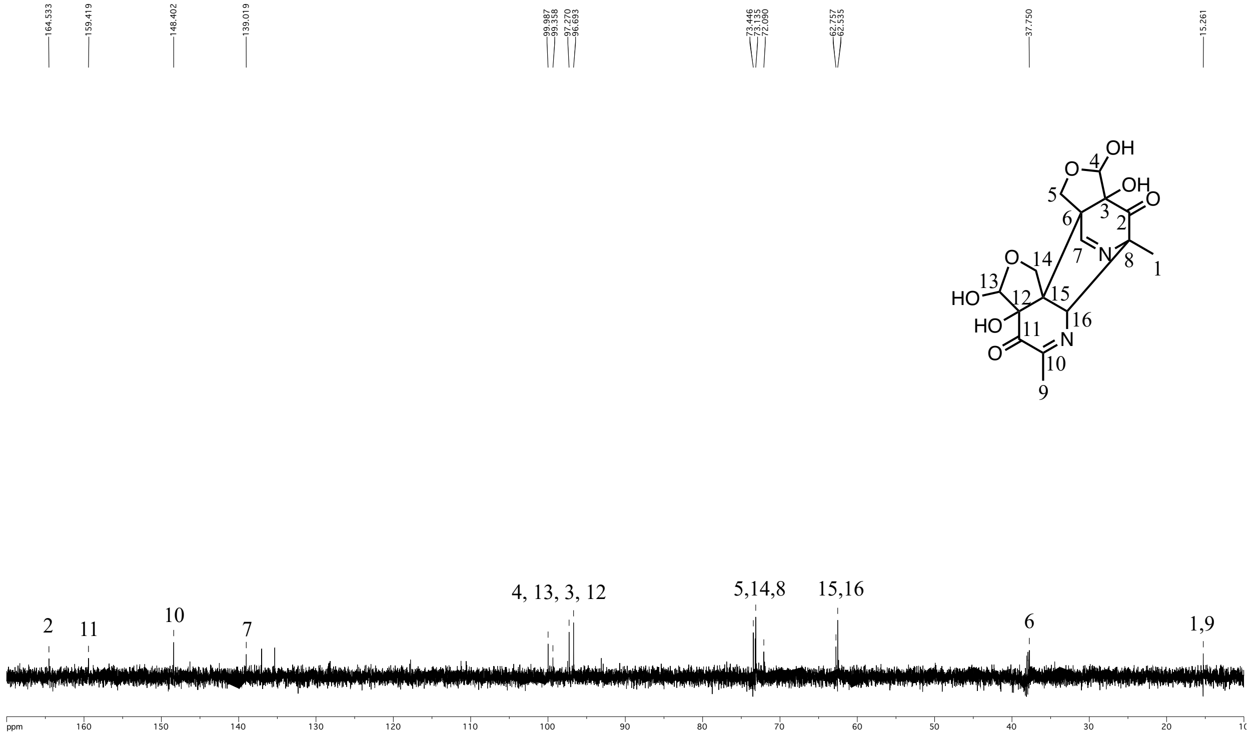

Figure S8. ^{13}C NMR Spectrum (125 MHz, D_2O) for **31**.

Enzymatic reaction condition for $P_{7.8}$ formation

The reaction conditions for $P_{7.8}$ formation were the same as those used for the THI5p reconstitution reaction described above. HPLC conditions 3 and 4 were used for the analysis of $P_{7.8}$ formation, isolation, and purification. LC-MS analysis (LC-MS condition 1) was carried out after dephosphorylation by CIP treatment of the reaction mixture.

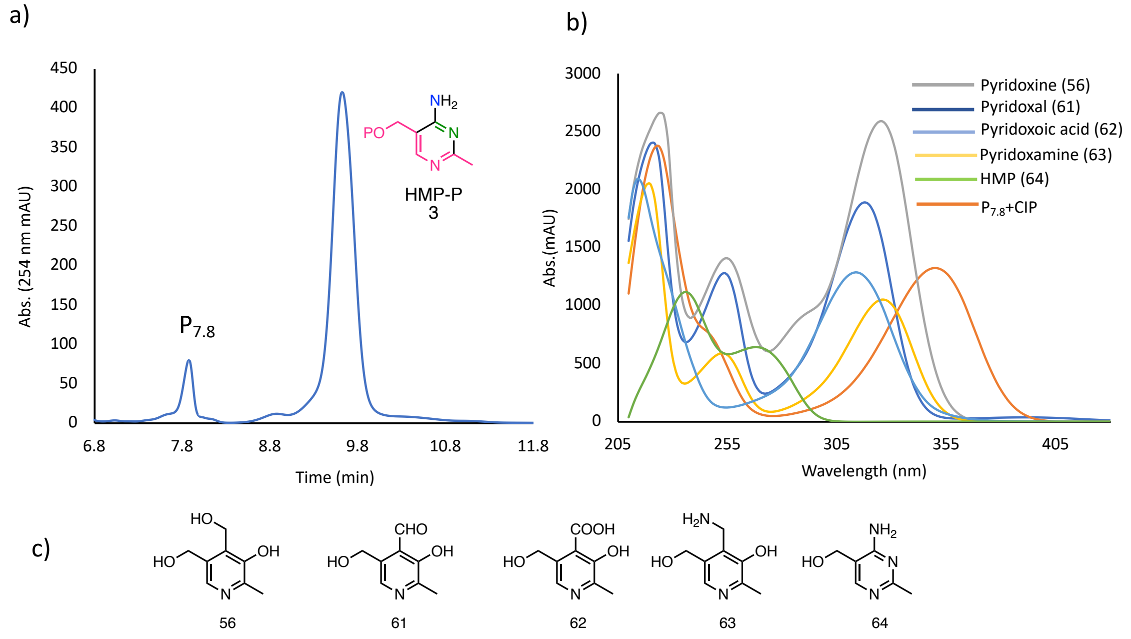

Figure S9. Identification of the shunt metabolite in the native THI5p reaction. a) HPLC chromatogram showing the formation of HMP-P and the $P_{7.8}$ metabolite in the native THI5p catalyzed reaction. b) Comparison of the UV-Vis spectra of CIP treated $P_{7.8}$ with pyridoxal, pyridoxal analogs and HMP. c) Structures of all compounds used for the UV-Vis comparison.

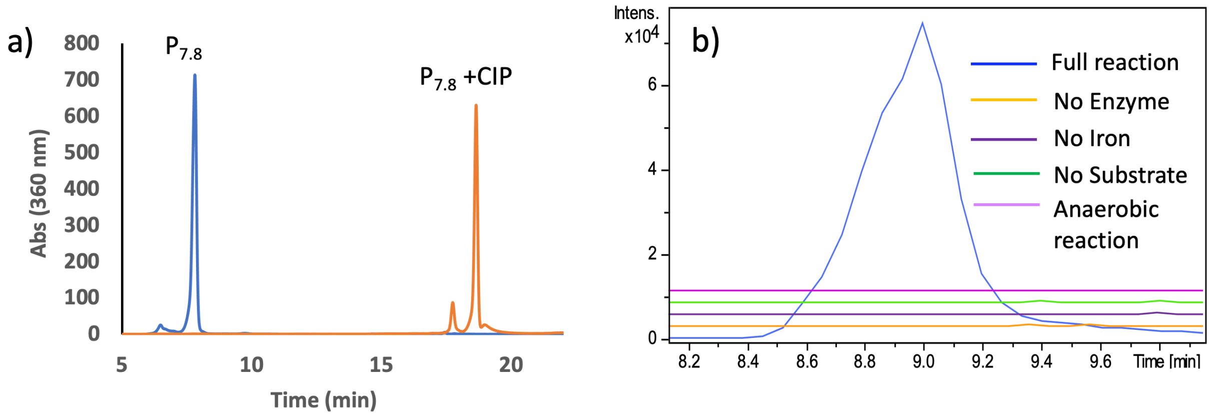

Figure S10. Dephosphorylation of $P_{7.8}$ and LC-MS analysis. a) Dephosphorylation (CIP treatment) of $P_{7.9}$ resulted in the formation of another new product ($P_{18.5}$) indicating the presence of a phosphate group. b) EIC of m/z 165.0659 (after dephosphorylation) in THI5p full reactions and other controls showing the shunt metabolite is formed only in a complete enzymatic reaction.

$P_{7.8}$ formation from isotopically labeled THI5p

The reaction conditions for $P_{7.8}$ formation were the same as those used for the THI5p reconstitution reaction described above except for the replacement of THI5p with ^{13}C-^{15}N- or ^{15}N-labelled protein. LC-MS analysis (condition 1) of the reactions showed a 1 Da increase in the mass of $P_{7.8}$ (from 165.0664 Da to 166.0629 Da).

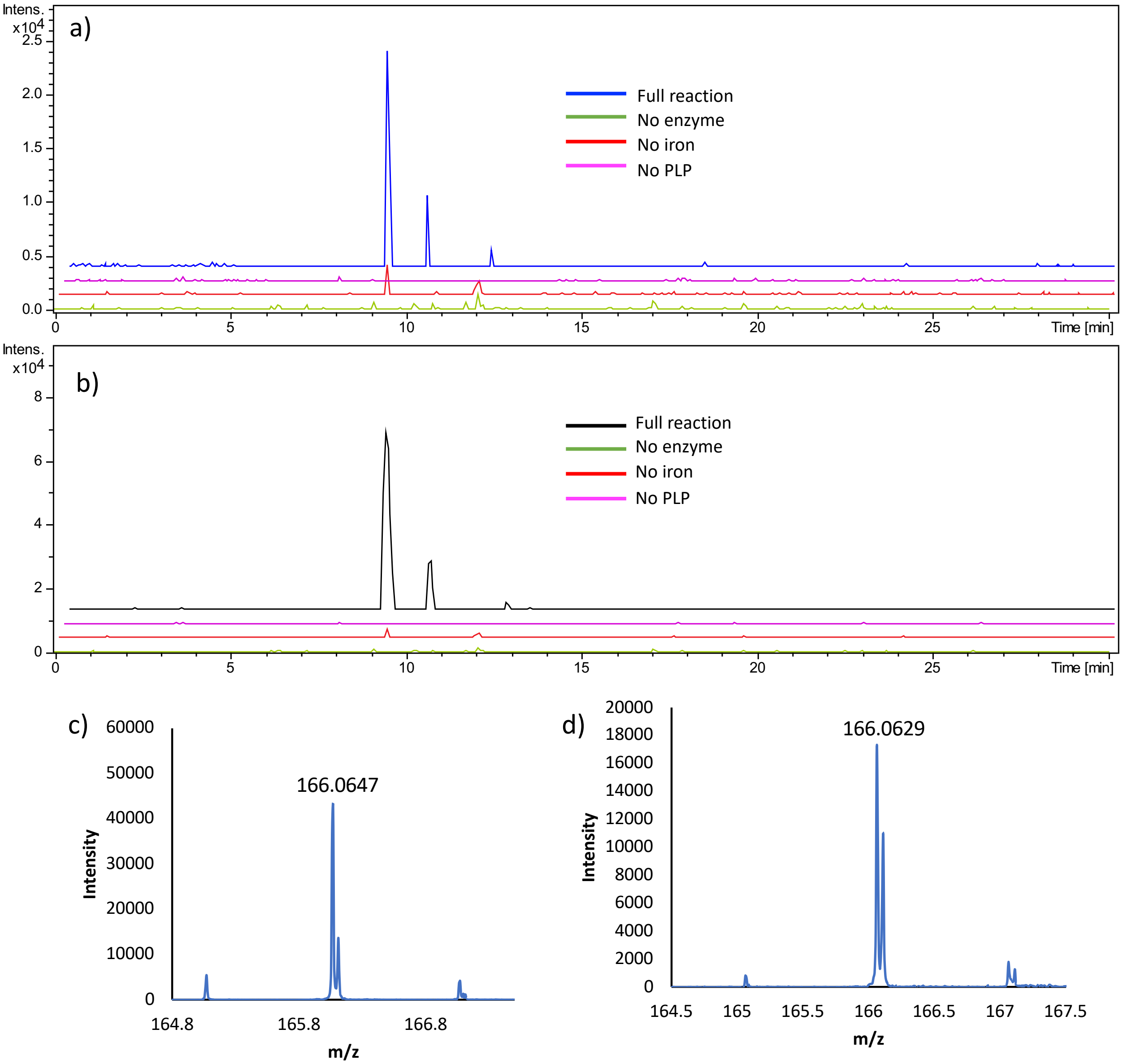

Figure S11. LC-MS analysis of $P_{7.8}$ formation with isotopically labeled THI5p. a) EIC of mass 166.0629 in ^{13}C-^{15}N THI5p full reaction along with controls. b) EIC of mass 166.0629 in the ^{15}N THI5p full reaction along with controls. c) and d) Mass spectra of $P_{7.8}$ (after dephosphorylation) obtained in ^{13}C-^{15}N THI5p full reaction and ^{15}N-THI5p full reactions respectively.

Synthesis of 34 (Cyano-Pyridoxal)

Pyridoxal (**61**, 1.0 mmol, 1.0 equiv.) and NH_2SO_3H (1.1 mmol, 1.1 equiv.) were dissolved in 4 mL of H_2O containing acetic acid (1.0 mmol, 1 equiv.) in a 10 mL round bottom flask at 0 °C. The reaction was stirred at 50°C until complete conversion by TLC analysis (5 hour).[5] The reaction was neutralized with aqueous 10% $NaHCO_3$ and lyophilized. The lyophilized product was HPLC purified (HPLC condition 4) The product was characterized by NMR and mass spectroscopy (very small amount of impurities present) and used for co-migration studies with dephosphorylated $P_{7.8}$ (HPLC condition 3). Mass in positive mode 165.0648 Da, ^{1}H NMR (400 MHz, DMSO-d_6): δ 7.237 (s, 1H), 4.359 (s, 2H), 2.181 (s, 3H). ^{13}C NMR (100 MHz, DMSO-d_6): δ 165.529, 151.473, 135.645, 126.197, 118.528, 99.878, 63.054, 22.052.

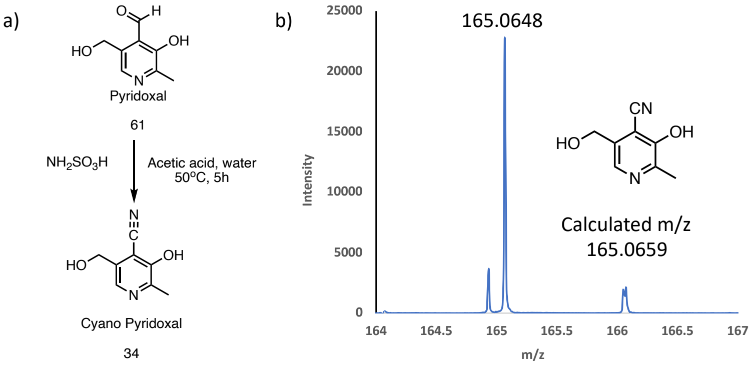

Figure S12. Synthesis of **34**. a) Synthetic scheme for the preparation of **34**. b) Mass spectrum of purified **34**.

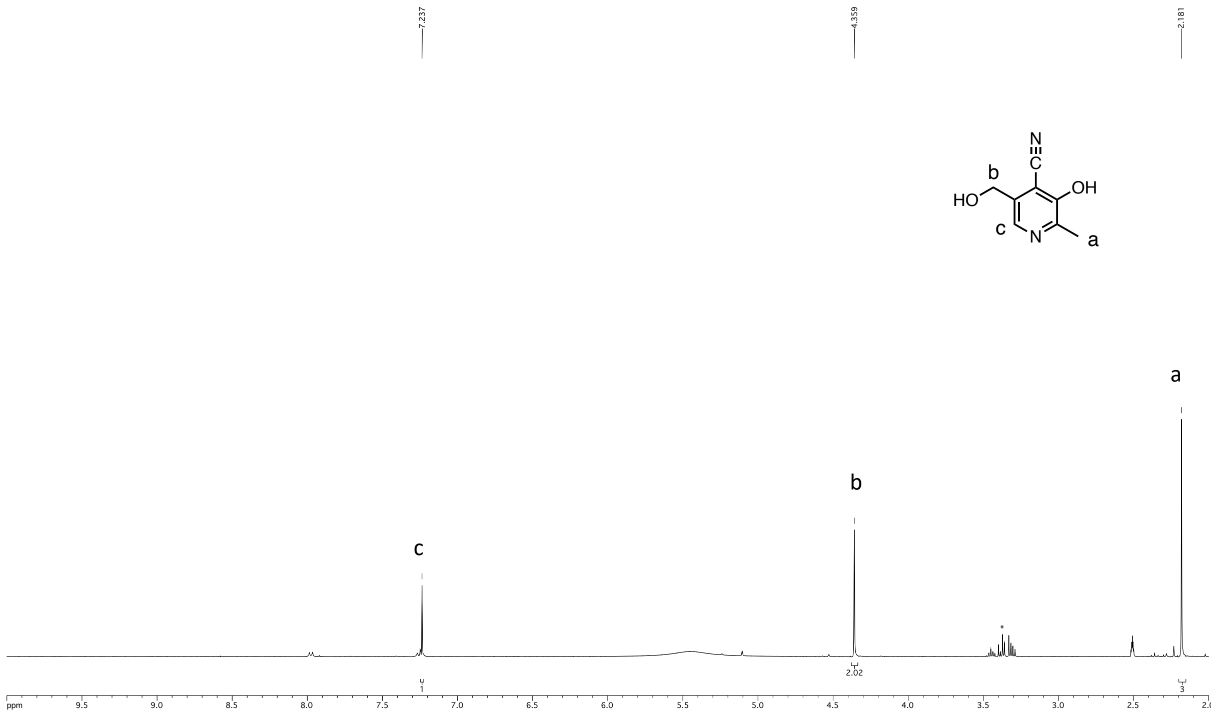

Figure S13. ^{1}H NMR spectrum of **34** (400 MHz, DMSO-d_6). The asterisks indicate minor impurities.

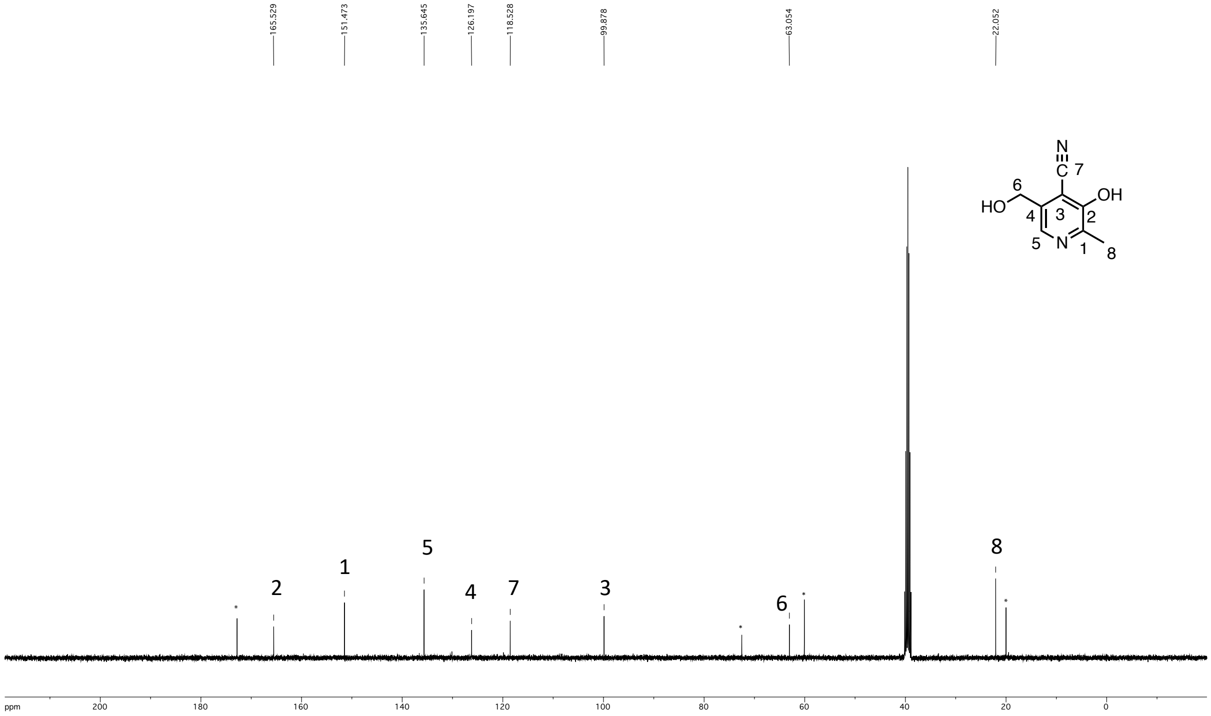

Figure S14. ^{13}C NMR spectrum of **34** (100 MHz, DMSO-*d*$_6$). The asterisks indicate minor impurities.

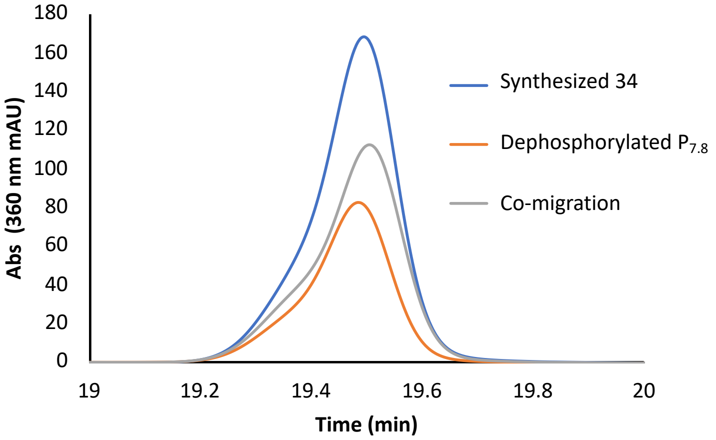

Figure S15. Co-migration of cyano-pyridoxal (dephosphorylated $P_{7.8}$ from native THI5p reaction) with synthetic standard **34**.

Quantitation of Cyano-PLP (33) formed in the THI5p reaction

For the quantitation of cyano-PLP a calibration curve was made by plotting peak area vs. concentration (HPLC condition 1) of synthesized cyano-pyridoxal. Then the peak area of the cyano-pyridoxal (~800 AU) from the THI5p reaction (obtained after dephosphorylation/CIP treatment) was fit to the equation derived from the calibration curve. In this way, it was determined

that 65 μM of cyano-PLP is formed which is ~ 27% of the enzyme concentration (240 μM THI5p was used) in the reaction (protein concentration was determined by absorbance measurement, the extinction coefficient of the protein was calculated as 50,310 $M^{-1}cm^{-1}$ using Protparam tool from Expasy (https://web.expasy.org/protparam/).

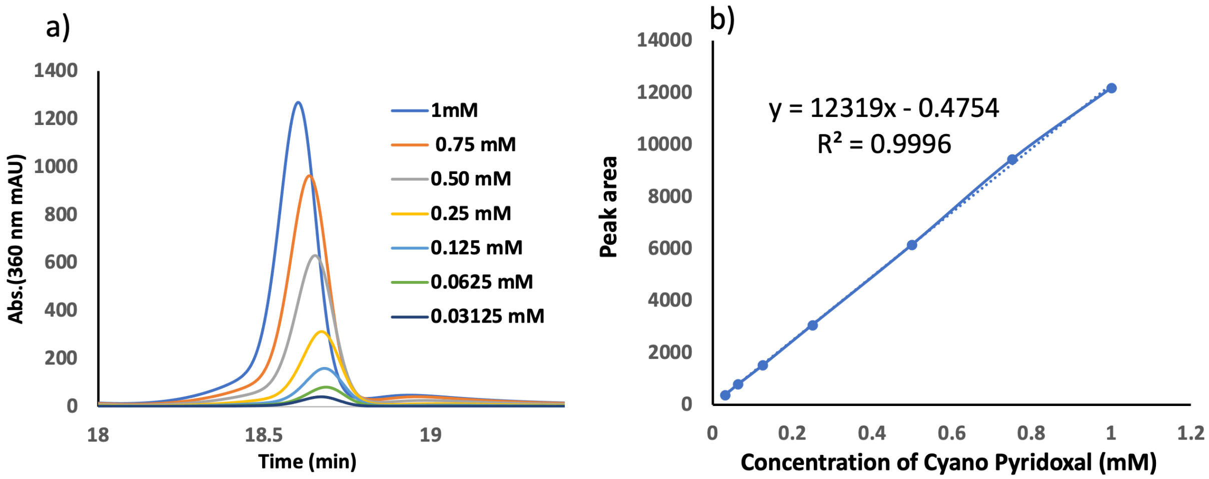

Figure S16. Quantitation of Cyano PLP in native THI5p reaction. a) HPLC chromatogram of synthesized cyano-pyridoxal standard in different concentrations ranging from 1 mM to 0.03 mM B) Calibration curve obtained by plotting peak area vs. concentration of Cyano-pyridoxal for the quantification of Cyano-PLP.

Conditions for trypsin digestion of single-turnover inactivated THI5p

The THI5p reaction mixture was desalted using a Bio-Spin 6 column (Bio-Rad) pre-equilibrated with 25 mM ammonium bicarbonate, pH 8.0. The inactive enzyme (50 μg, 1.8 μL of 660 μM THI5p) was added to 10 μL of 6M guanidine-HCl, 25 mM ammonium bicarbonate, pH 8.0. DTT (1 μL of 200 mM solution in 25 mM ammonium bicarbonate, pH 8.0) was added and the resulting mixture was incubated at room temperature for 1 hour. After incubation 10 μL of 200 mM iodoacetamide in 25 mM ammonium bicarbonate, pH 8.0, was added and the reaction mixture was incubated at room temperature in the dark for 1 hour. Finally, 77.5 μL of 25 mM ammonium bicarbonate, pH 8.0 was added to dilute the guanidine-HCl to 0.6M followed by addition of 1 μg of trypsin. The mixture was incubated at 37°C overnight and stored at -20°C until analysis (LC-MS condition 2).[6-8]

Conditions for trypsin digestion of methoxyamine-derivatized THI5p (38)

After the completion of the THI5p reaction (carried out in presence of catalase, THI5p: catalase = 1:1000), excess methoxyamine hydrochloride (2 μL of 1.2 M solution) was added and the reaction mixture was incubated at 37°C for 2 hours and desalted using a Bio-Spin 6 column (Bio-Rad) pre-equilibrated with 25 mM ammonium bicarbonate, pH 8.0. The inactive enzyme (50 μg, 1.8 μL of 660 μM THI5p) was added to 10 μL of 6M guanidine-HCl, 25 mM ammonium bicarbonate, pH 8.0. After DTT addition (1 μL of 200 mM DTT in 25 mM ammonium bicarbonate, pH 8.0) the resulting mixture was incubated at room temperature for 1 hour. Iodoacetamide (10 μL of 200 mM solution in 25 mM ammonium bicarbonate, pH 8.0) was then added and the reaction mixture was incubated at room temperature in the dark for 1 hour. Finally, 77.5 μL of 25 mM ammonium bicarbonate, pH 8.0 was added to dilute the guanidine-HCl to 0.6M followed by

addition of 1 µg of trypsin. The mixture was incubated at 37°C overnight and stored at -20°C until analysis (LC-MS condition 2).

a)

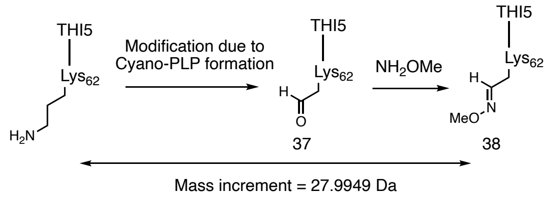

b) Obtained peptide fragment with modification

^{57}VDMGLK*AMVHTLAAK^{71}

Calculated mass for the unmodified peptide–1584.8600 Da
Obtained mass for the modified peptide – 1612.8546 Da
Calculated mass for the modified peptide – 1612.8549 Da

Obtained difference = 27.9946 Da
Predicted difference = 27.9949 Da
Accuracy – 0.18 ppm

c)

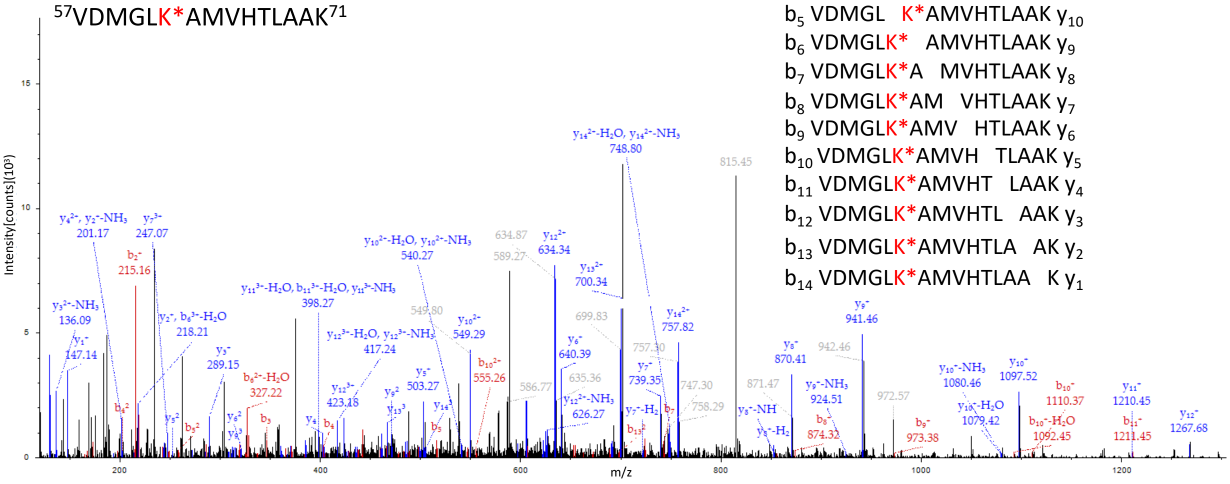

b_1 V DMGLK*AMVHTLAAK y_{14}
b_2 VD MGLK*AMVHTLAAK y_{13}
b_3 VDM GLK*AMVHTLAAK y_{12}
b_4 VDMG LK*AMVHTLAAK y_{11}
b_5 VDMGL K*AMVHTLAAK y_{10}
b_6 VDMGLK* AMVHTLAAK y_9
b_7 VDMGLK*A MVHTLAAK y_8
b_8 VDMGLK*AM VHTLAAK y_7
b_9 VDMGLK*AMV HTLAAK y_6
b_{10} VDMGLK*AMVH TLAAK y_5
b_{11} VDMGLK*AMVHT LAAK y_4
b_{12} VDMGLK*AMVHTL AAK y_3
b_{13} VDMGLK*AMVHTLA AK y_2
b_{14} VDMGLK*AMVHTLAA K y_1

Figure S17. MS/MS analysis of **38**. a) Mass increment for the conversion of the Lys62-containing peptide to **38** is 27.9949 Da. b) Calculation of the mass increment. c) MS-MS fragmentation of the modified peptide **38**. This fragmentation pattern is consistent with the predicted aldehyde modification on Lys62. The fragmented peptide sequence for b and y ions are also shown. Modified Lys 62 residue is indicated in red as K*.

Enzymatic reaction condition for chemical rescue experiment with THI5p-H66G

The chemical rescue experiment was performed with the THI5p-H66G mutant. The reaction was performed in 100 mM HEPES buffer with 1mM TCEP, pH 7.5 containing THI5p-H66G (240 µM), $Fe(NH_4)_2(SO_4)_2$ (120 µM), PLP (1200 µM) and imidazole (10 to 2000 mM).[9-13] The enzyme was anaerobically preincubated with $Fe(NH_4)_2(SO_4)_2$ in an ice bath for 30 min. Then PLP was added, and the reaction mixture was incubated for another 30 minutes at room temperature. Imidazole was then added, and the reaction was aerobically incubated at room temperature for 4 hr. The reaction was quenched by filtering with a 10 kDa cut-off filter and analyzed by HPLC (condition 1) and LC-MS (condition 1, LC-MS was done after dephosphorylation by CIP treatment of the sample).

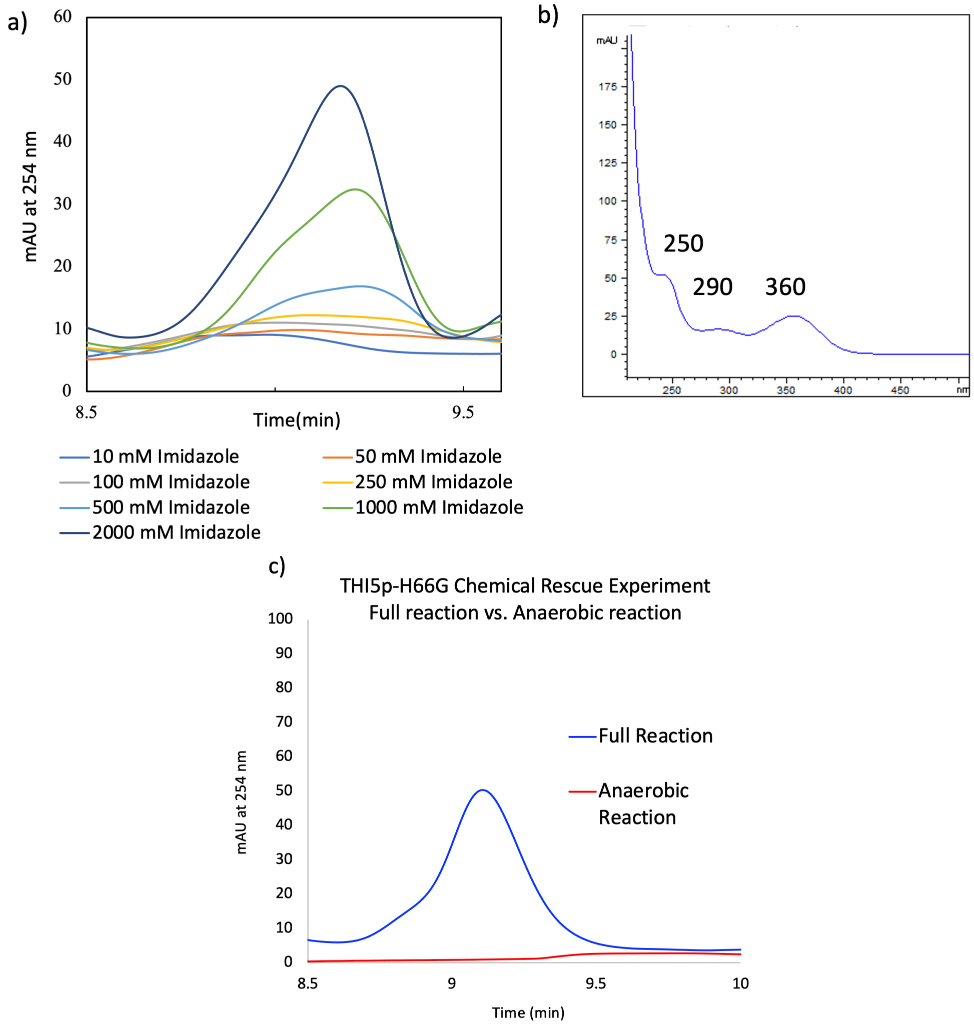

Figure S18. HPLC analysis of THI5p-H66G reaction. a) Imidazole concentration dependence of the formation of $P_{9.2}$. b) UV spectrum of $P_{9.2}$. c) HPLC chromatogram for the anaerobic control (reaction run in absence of oxygen) of the THI5p-H66G reaction showing that formation of $P_{9.2}$ requires oxygen.

Dephosphorylation of the THI5p-H66G reaction product

After completion of the THI5p-H66G-catalyzed reaction, the reaction mixture was treated with CIP (20 units) and incubated at 37ºC for 2 hr. Then the mixture was quenched by filtering with a 10 kDa cut-off filter and analyzed by HPLC (condition 1) and LC-MS (condition 1).

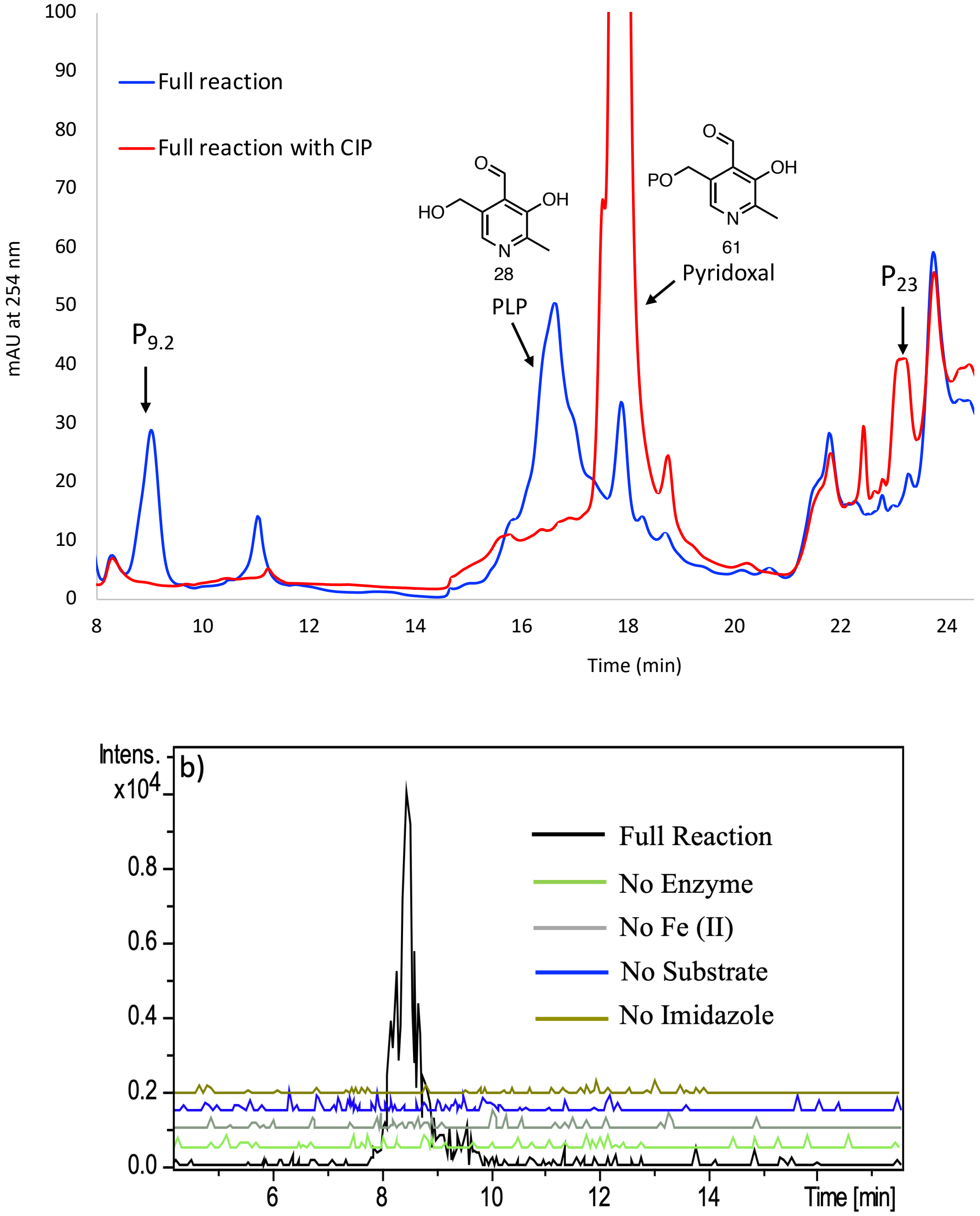

Figure S19. Dephosphorylation and LC-MS analysis of the THI5p-H66G reaction. a) Dephosphorylation of the THI5p-H66G reaction product $P_{9.2}$ to give P_{23}. b) Extracted Ion Chromatograms (EIC) of the mass 250.0833 in THI5p-H66G reaction (after dephosphorylation) showing the formation of dephosphorylated $P_{9.2}$ in the full reaction only.

$^{18}O_2$ labeling of $P_{9.2}$

The chemical rescue reaction was carried out as described above except $^{16}O_2$ was replaced with $^{18}O_2$.

2-D-imidazole labeling of $P_{9.2}$

The chemical rescue reaction was carried out as described above except imidazole was replaced with 2-D-imidazole.[14]

2'-CD_3-PLP and 4'-formyl-D-2'-CD_3-PLP labeling of $P_{9.2}$

The chemical rescue reaction was carried out as described above except PLP was replaced by a synthetic mixture of 2'-CD_3-PLP and 4'-formyl-D-2'-CD_3-PLP.[1, 15]

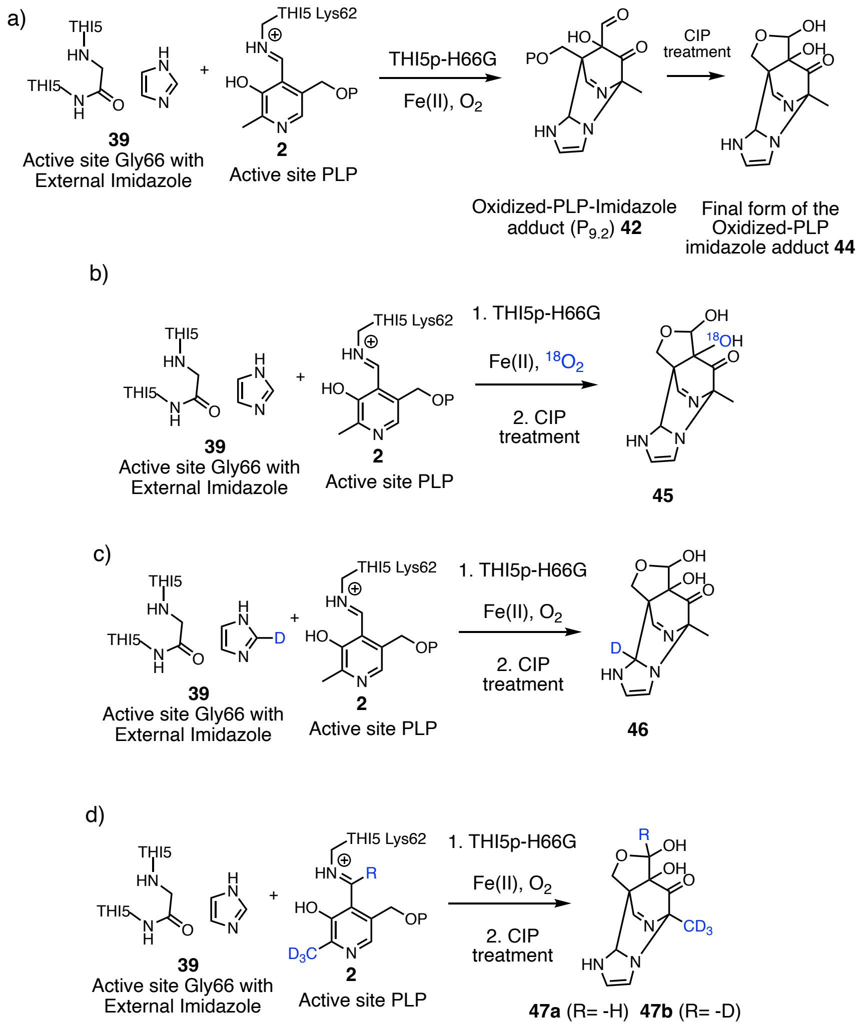

Figure S20. Characterization of the dephosphorylated PLP imidazole reaction product (dephospho-$P_{9.2}$ or P_{23}). a) Proposed formation of dephospho-$P_{9.2}$ (**44**). b) Formation of dephospho-$P_{9.2}$ using $^{18}O_2$. c) Formation of dephospho-$P_{9.2}$ using 2-D-imidazole. d) Formation of dephospho-$P_{9.2}$ using a mixture of 2'-CD_3-PLP and 4'-formyl-D-2'-CD_3-PLP.

Synthesis of 2-D-imidazole

2-D-imidazole was synthesized according to the procedure reported by Proniewicz *et al.*[14]

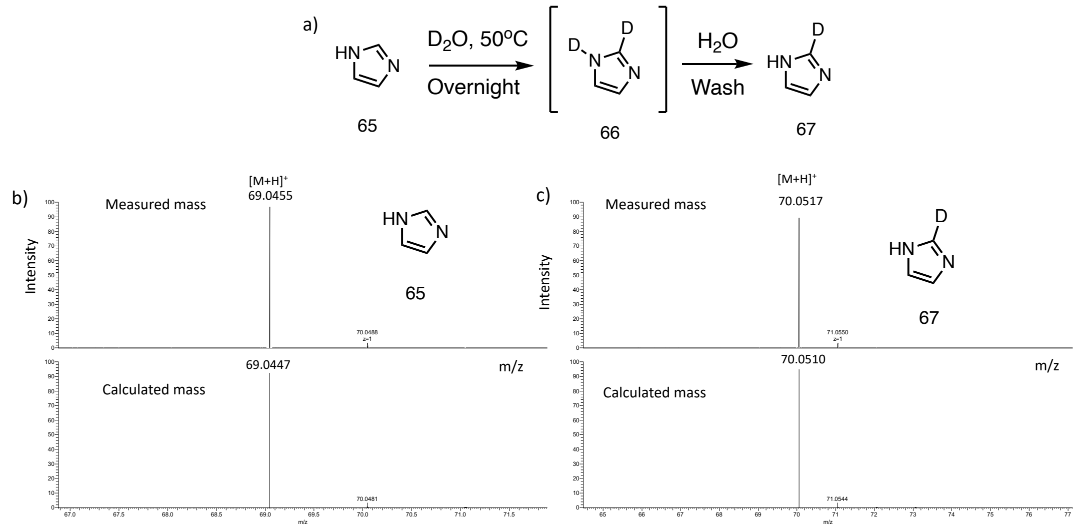

Figure S21. Synthesis of 2-D-imidazole. a) Synthetic scheme for the synthesis of 2-D-imidazole. b) and c) Mass of natural abundance imidazole **65** and synthesized 2-D-imidazole **67**.

Synthesis of deuterated Pyridoxal Phosphate (mixture of 2'-CD_3-PLP and 4'-formyl-D-2'-CD_3-PLP)

Deuterated pyridoxal was synthesized, with some minor modifications, as previously reported (Figure S22).[1, 15] For the enzymatic phosphorylation, 10 µL of 10 mM pyridoxal was added to 5 µL 60 µM pyridoxal kinase (PdxK) in 53 µL of 100 mM HEPES buffer (pH 7.5). Then 20 µL of 10 mM ATP and 2 µL of 0.1M $MgSO_4$ were added. The reaction mixture was incubated at 37°C for 5 hours and then filter quenched with 10 kDa cut-off filter. Labeled PLP was purified by HPLC (Condition 2) as a mixture of **72a** and **72b**. After HPLC purification, for LC-MS analysis (condition 1) they were dephosphorylated to improve the flying efficiency.

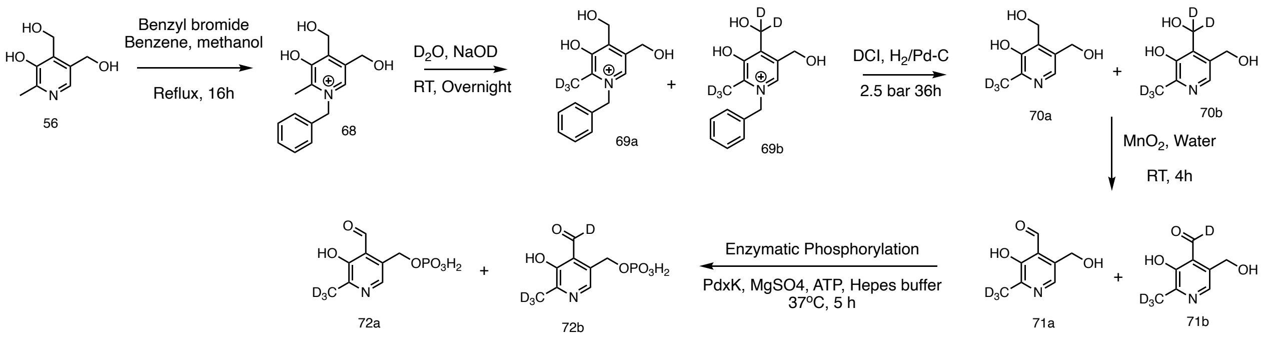

Figure S22. Synthetic scheme for the preparation of deuterated PLPs (mixture of **72a** and **72b**).

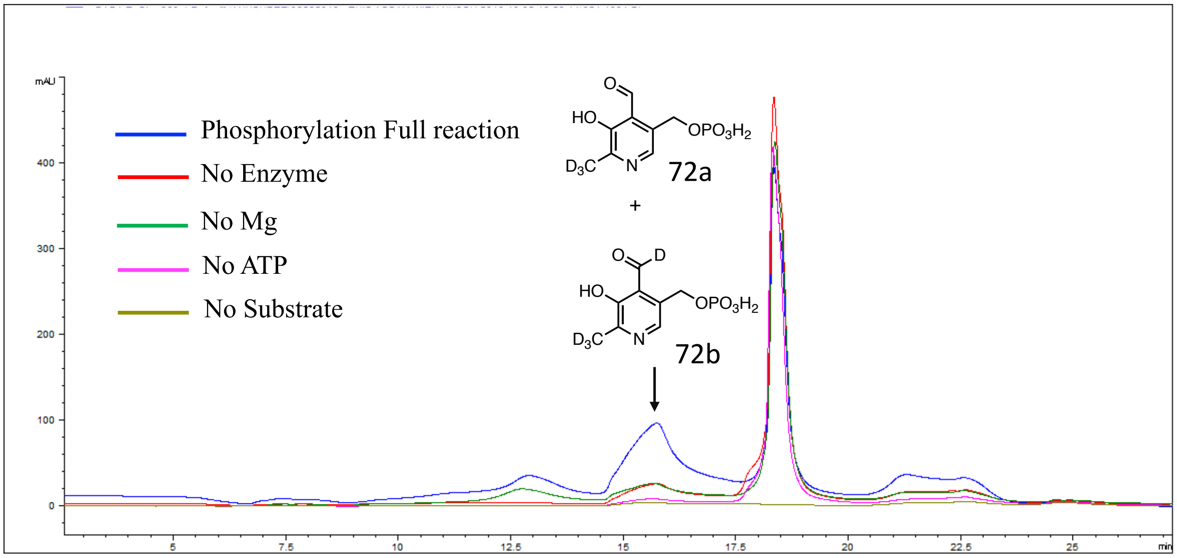

Figure S23. HPLC chromatogram at 360 nm of the pyridoxal (**71a** and **71b**) phosphorylation reaction mixture indicating the formation of **72a** and **72b**.

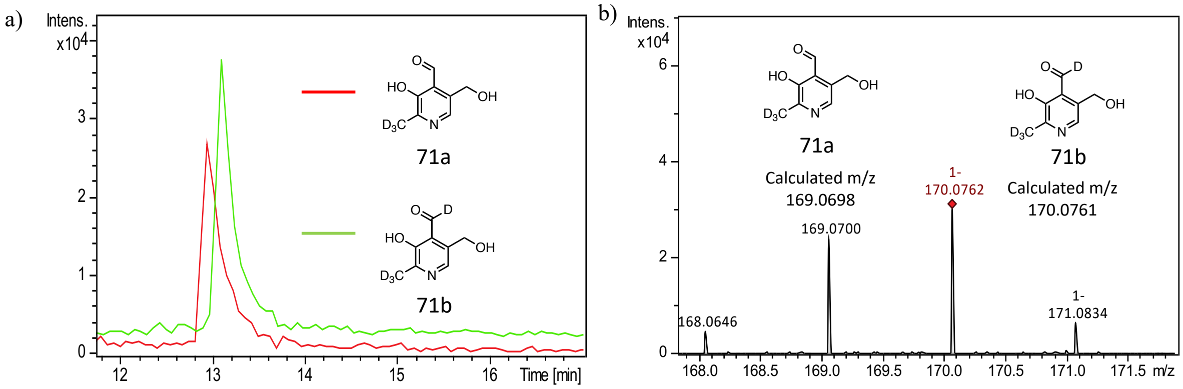

Figure S24. LC-MS analysis of the mixture of **72a** and **72b** after HPLC purification and dephosphorylation (by CIP). a) Extracted ion chromatograms of deuterated pyridoxal phosphates (**72a** and **72b**) after HPLC purification and dephosphorylation in the form of **71a** and **71b**. b) Mass spectrum of pyridoxal phosphates after HPLC purification and dephosphorylation.

Synthesis of PLP-oxime 48

PLP oxime **48** was synthesized as previously described,[16] purified by HPLC (condition 2) and analyzed, after dephosphorylation, by LC-MS (condition 1). Mass in positive mode after dephosphorylation is 183.0792. ^{1}H NMR (400 MHz, D_2O): δ 8.597 (s, 1H), 7.923 (s, 1H), 5.030-5.013 (d, 2H, J=6.8 Hz), 2.484 (s, 3H). ^{13}C NMR (100 MHz, D_2O): δ 147.830, 146.394, 131.915, 131.832, 124.613, 99.981, 62.005, 16.432.

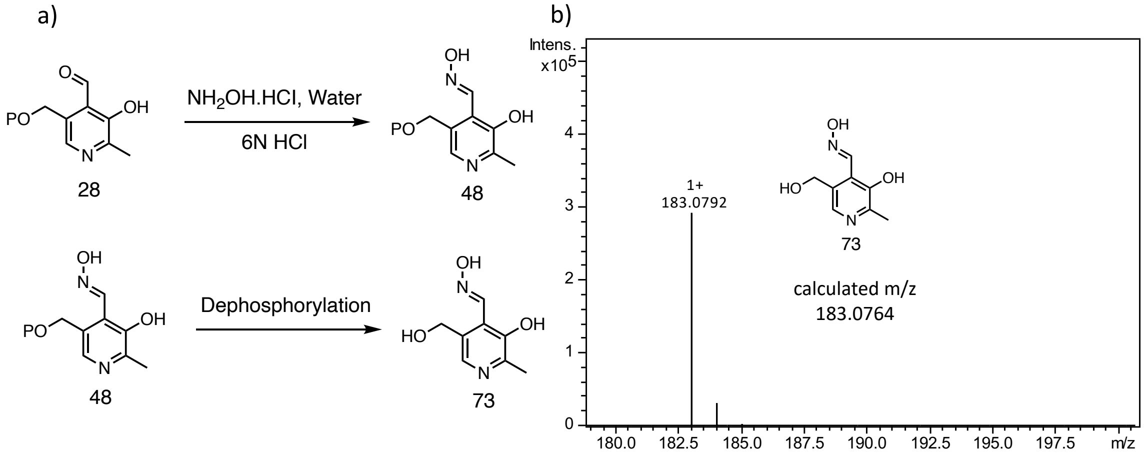

Figure S25. Synthesis of **48**. a) Synthetic scheme for the preparation and dephosphorylation of **48**. b) Mass spectrum of dephosphorylated **48**.

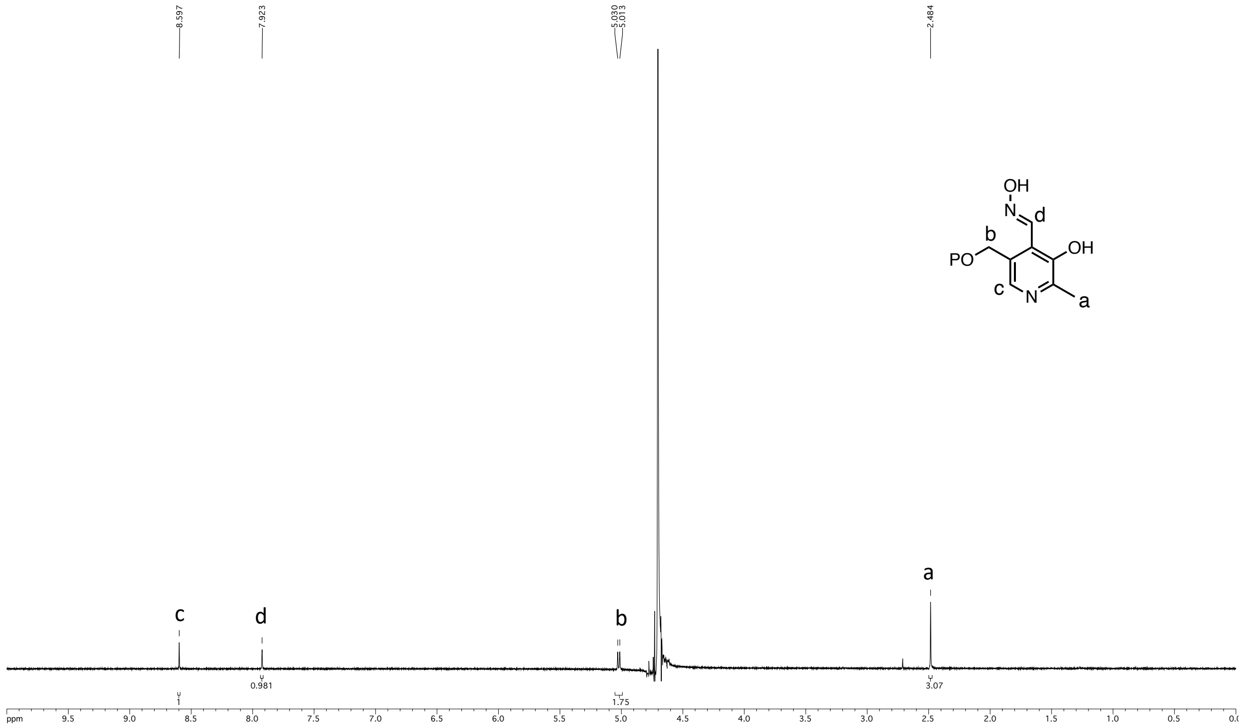

Figure S26. ^{1}H NMR spectrum of PLP-oxime **48** (400 MHz in D_2O).

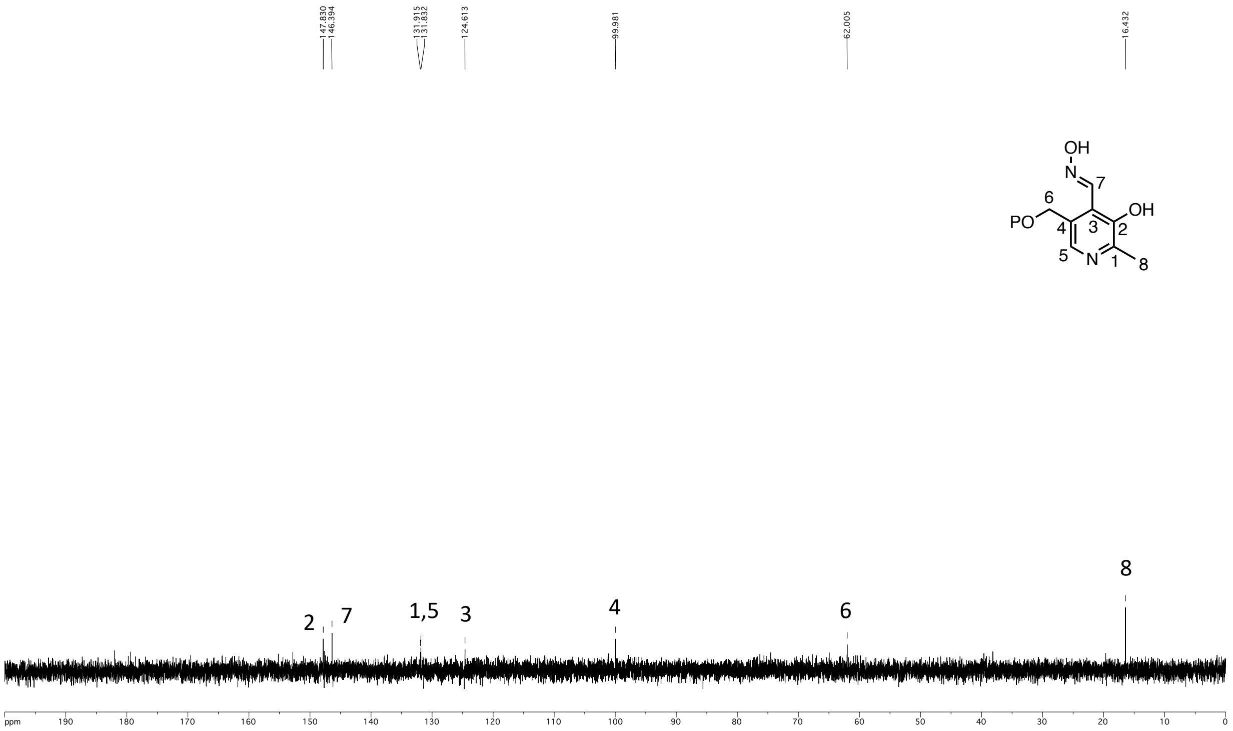

Figure S27. ^{13}C NMR spectrum of PLP-oxime **48** (100 MHz in D_2O).

Conditions for the THI5p-K62A-catalyzed reaction

The THI5p-K62A reaction was performed in 100 mM HEPES buffer with 1mM TCEP, pH 7.5 containing THI5p (240 µM), $Fe(NH_4)_2(SO_4)_2$ (120 µM) and PLP-oxime (600 µM). The enzyme was anaerobically preincubated with $Fe(NH_4)_2(SO_4)_2$ in an ice bath for 30 min. Then PLP-oxime was added, and the reaction mixture was aerobically incubated at room temperature for 4 hr. The reaction was quenched by filtering with a 10 kDa cut-off filter and analyzed by HPLC (HPLC condition 3) and LC-MS (LC-MS (condition 1) was carried out after CIP treatment of the sample.

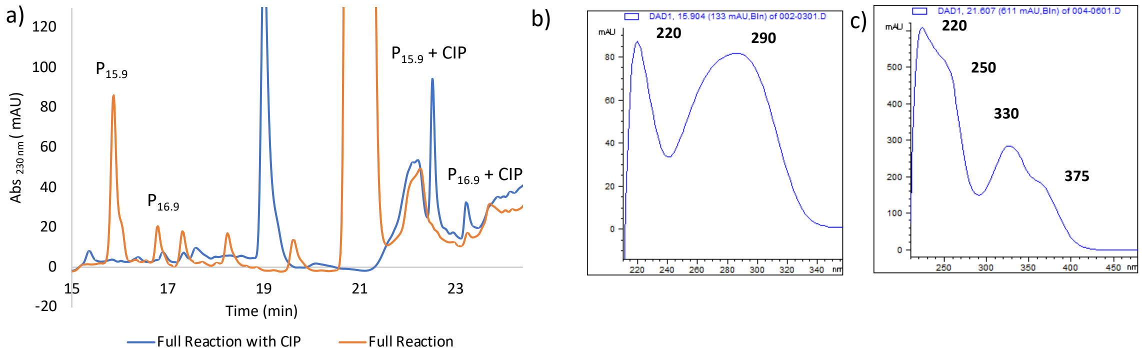

Figure S28. HPLC analysis of THI5p-K62A reaction. a) HPLC Chromatogram of THI6p-K62A reaction at 230 nm showing dephosphorylation (by CIP treatment) of $P_{15.9}$ and $P_{16.9}$. b) and c) UV-Vis spectra of $P_{15.9}$ and $P_{16.9}$.

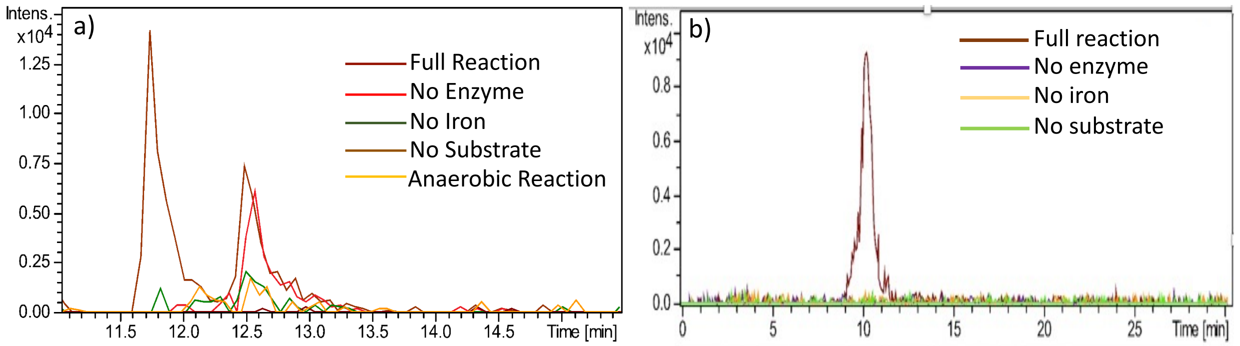

Figure S29. LC-MS analysis of THI5p-K62A reaction. Extracted Ion Chromatograms of dephosphorylated $P_{15.9}$ (a) for mass 181.0608 Da (retention time 11.5-12.0 min) and $P_{16.9}$ (b) for mass 199.0713 Da showing the formation of these products in THI5p-K62A full reaction.

Synthesis of 52

2'-Hydroxymethyl pyridoxal-oxime (**52**) was prepared as described in the synthetic scheme shown in Figure S30a.[17-19] After following the synthetic scheme and HPLC purification (condition 3), it was used as a reference for the identification of the dephosphorylated THI5p-K62A reaction product ($P_{16.9}$) (HPLC condition 3 and LC-MS (condition 1). Mass in positive mode 199.0724. ^{1}H NMR (400 MHz, DMSO-d_6): δ 8.557 (s, 1H), 7.987 (s, 1H), 4.583 (s, 4H). ^{13}C NMR (100 MHz, DMSO-d_6): δ 150.940, 148.742, 147.561, 138.120, 133.949, 121.643, 60.688, 59.487.

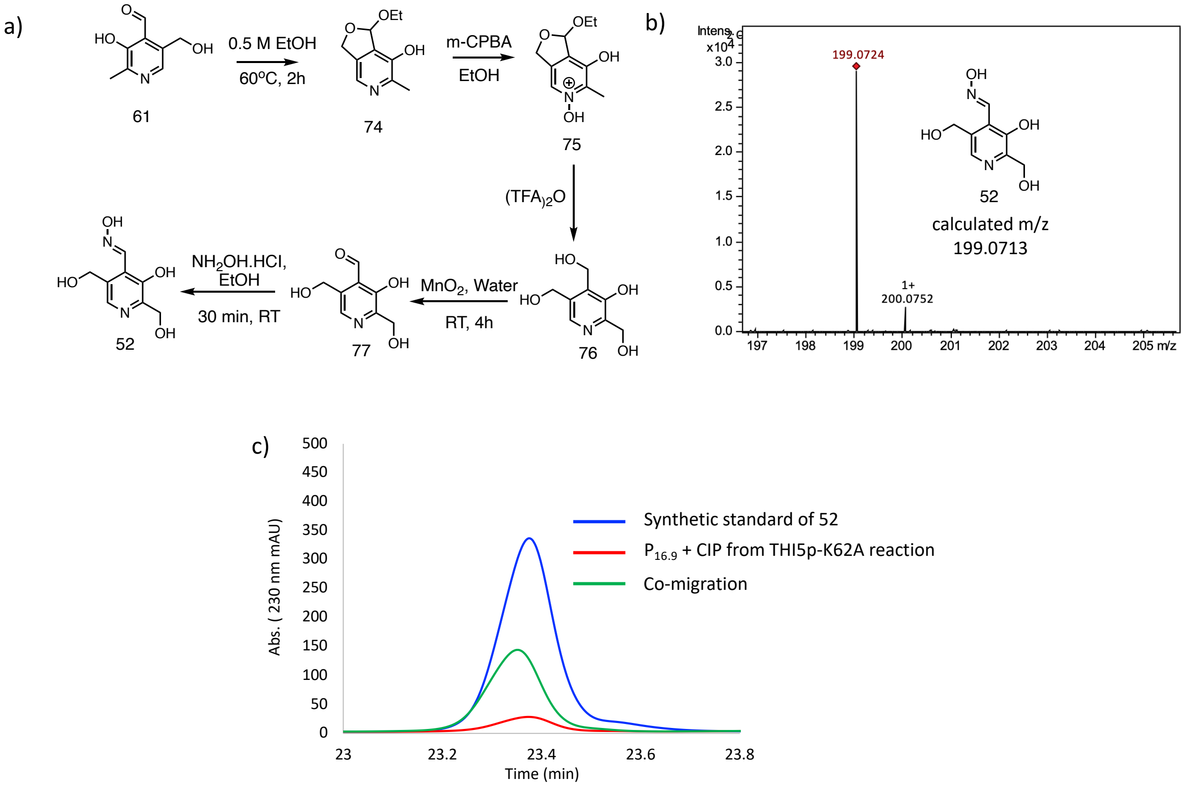

Figure S30. Synthesis of **52**. a) Synthetic scheme for the preparation of **52**. b) Mass of synthetic standard of **52**. c) HPLC chromatogram at 360 nm showing co-migration of dephosphorylated $P_{16.9}$ with **52**.

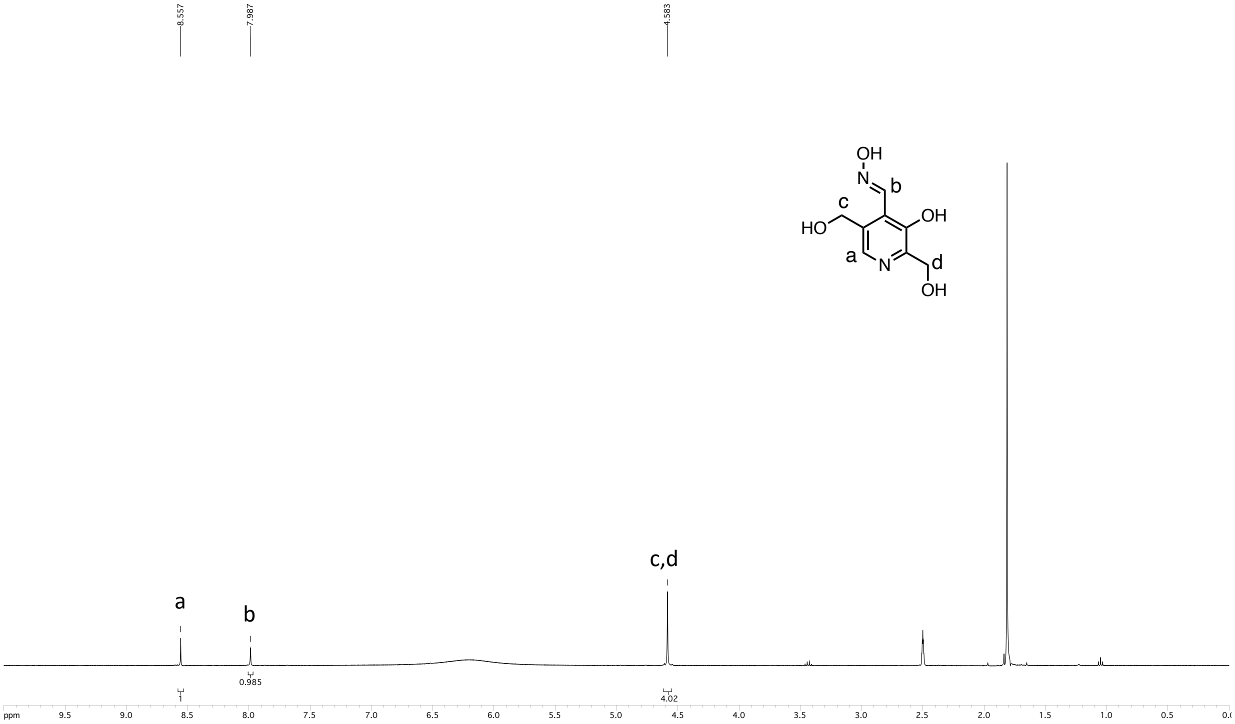

Figure S31. ^{1}H NMR spectrum of **52** (400 MHz in DMSO-d_6 (peak at 1.9 - acetate).

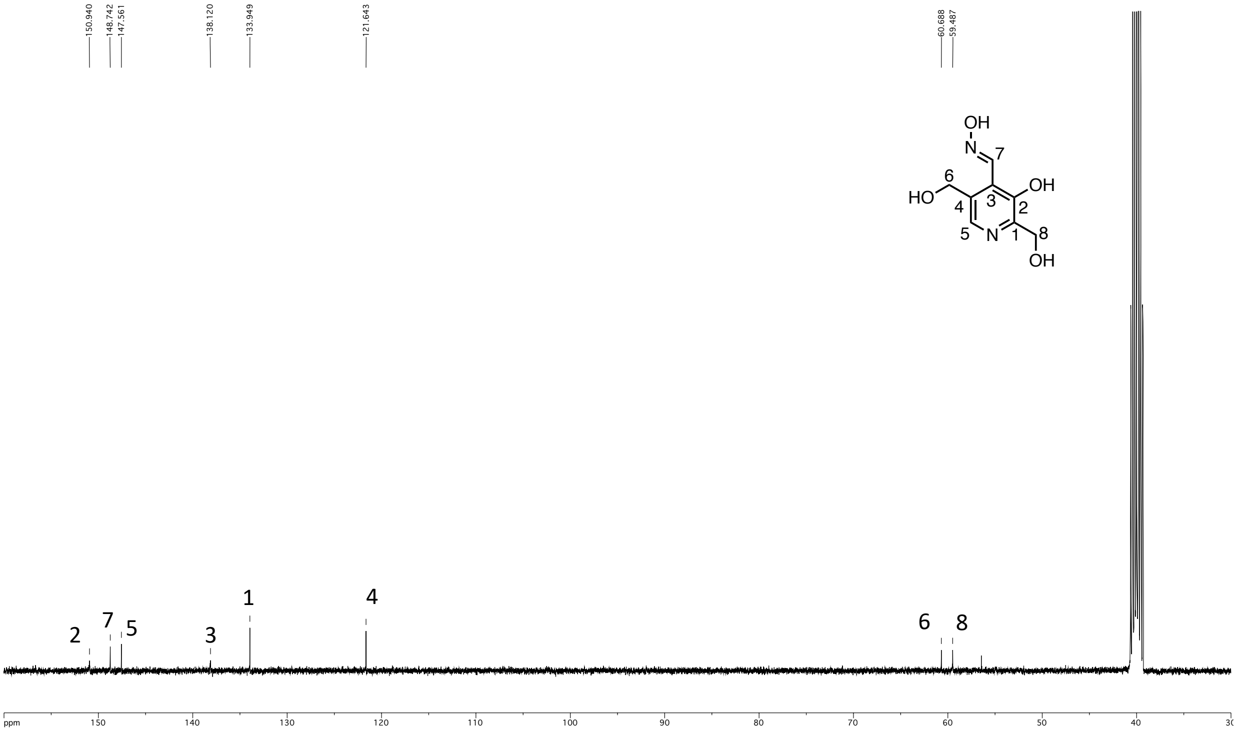

Figure S32. ^{13}C NMR spectrum of **52** (100 MHz in DMSO-d_6).

Characterization of $P_{15.9}$ from the THI5p-K62A catalyzed reaction

Pyridoxal nitrile oxide from the THI5p-K62A reaction was dephosphorylated and purified by HPLC (condition 4). It was derivatized by reaction with pyridoxal (**61**) in water to form the 1,3-dipolar reaction product (**78**). The reaction mixture was then heated at reflux in the presence of t-BuOK for 2 hours followed by neutralization and solvent removal.[20] The resulting reaction mixture containing cyano pyridoxal (**34**) and pyridoxoic acid (**62**) was analyzed by HPLC (condition 6) and LC-MS (condition 1) and used for the co-migration studies.[5]

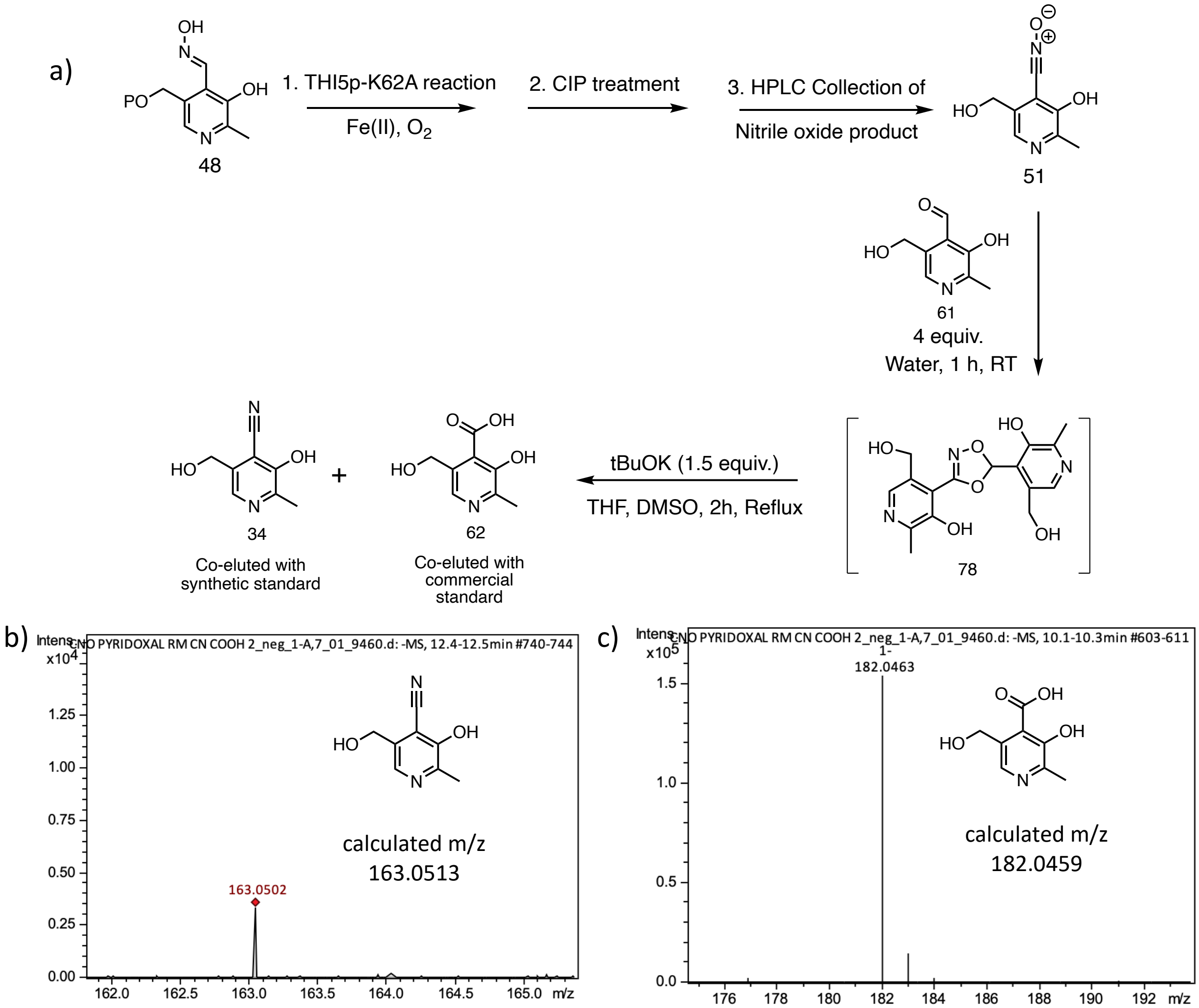

Figure S33. Characterization of $P_{15.9}$. a) Strategy for the characterization of $P_{15.9}$ by its conversion to **34**. b) and c) Mass spectra of the products obtained following scheme (a).

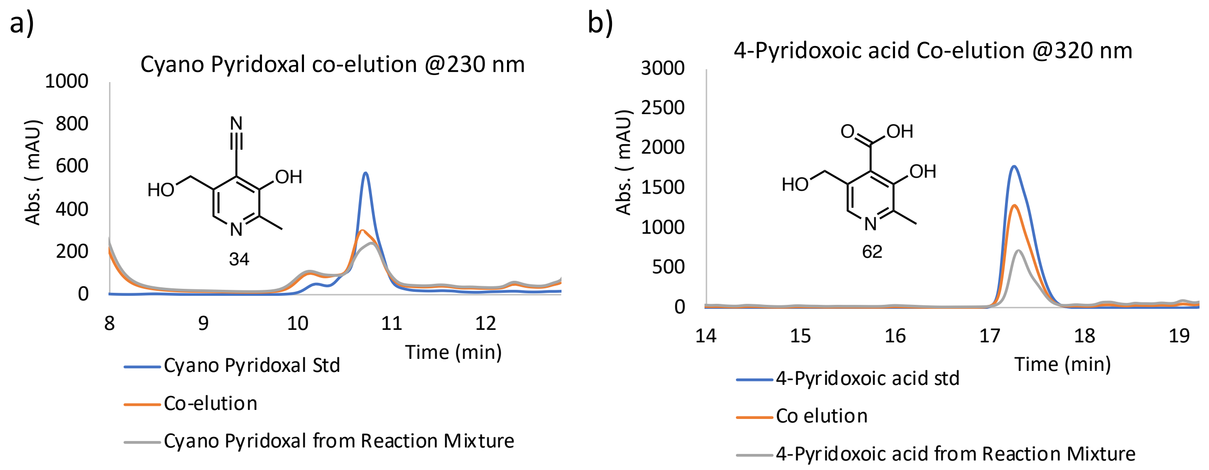

Figure S34. HPLC analysis of products formed following scheme (a) of Figure S33. a) HPLC chromatogram showing co-elution of the derivatized THI5p-K62A reaction product ($P_{15.9}$) with a synthesized sample of cyano-pyridoxal **34**. b) HPLC chromatogram showing co-elution of the pyridoxoic acid product obtained following reaction scheme S33a with a commercial standard.

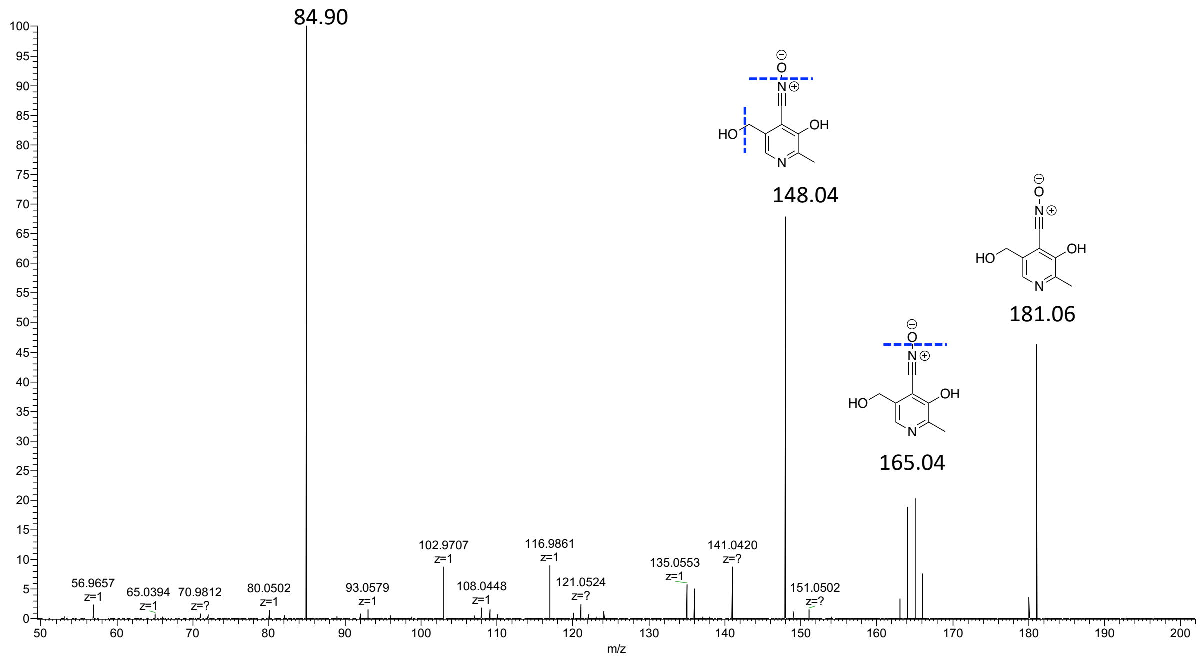

Figure S35. MS/MS fragmentation spectra of dephosphorylated and HPLC purified $P_{15.9}$ in positive mode showing additional support for its structural assignment.

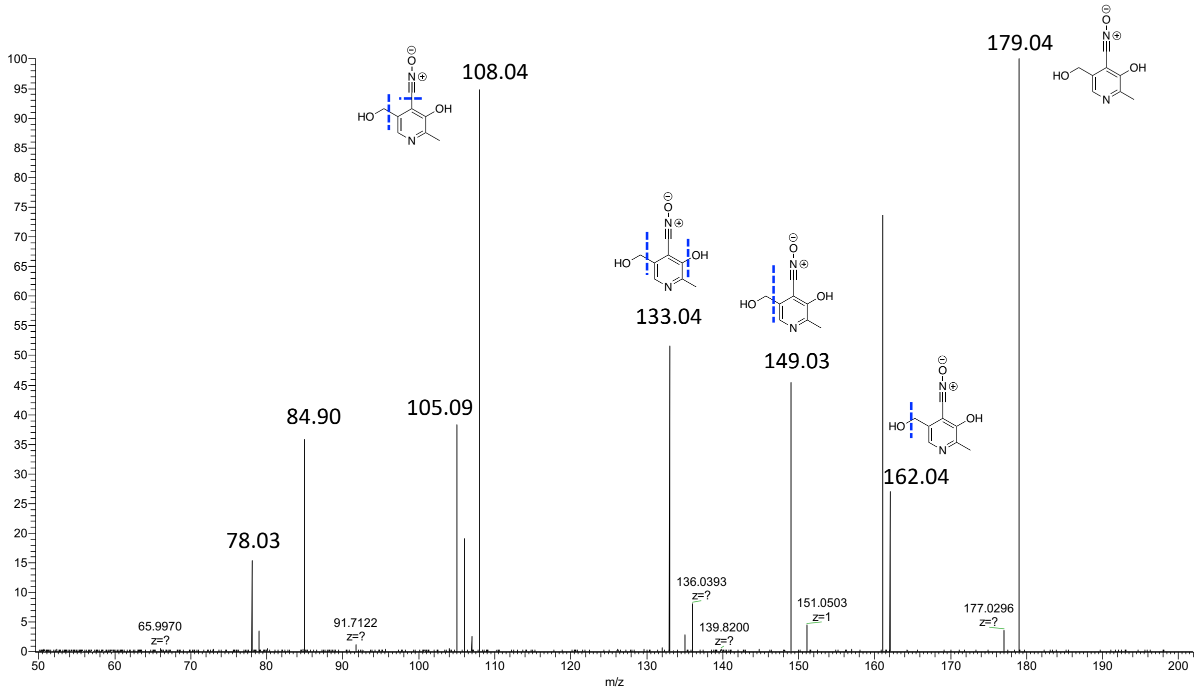

Figure S36. MS/MS fragmentation spectra of dephosphorylated and HPLC purified $P_{15.9}$ in negative mode showing additional support for its structural assignment.

Synthesis of 2'-CD_3- [48]

2'-CD_3-[**48**] was prepared following Scheme (a) in Figure S37.[15, 16] For the enzymatic phosphorylation, 10 μL of 10 mM pyridoxal was added to 5 μL 60 μM pyridoxal kinase in 53 μL of 100 mM HEPES buffer (pH=7.5). Then 20 μL of 10 mM ATP and 2 μL of 0.1M $MgSO_4$ were added. The reaction was incubated at 37°C for 5 hour and then filter quenched with a 10 kDa cut-off filter. The filtrate was treated with hydroxylamine as described above for the preparation of **48** and the resulting oxime was HPLC purified (condition 4) and analyzed by LC-MS (condition 1) after CIP treatment. Mass in positive mode after dephosphorylation is 186.0966 Da. ^{1}H NMR (400 MHz, D_2O): δ 8.674 (s, 1H), 7.963 (s, 1H), 5.038-5.024 (d, 2H, J=5.6 Hz). ^{13}C NMR (100 MHz, D_2O): δ 148.259, 146.573, 132.784, 130.953, 125.650, 119.046, 62.482, 13.425.

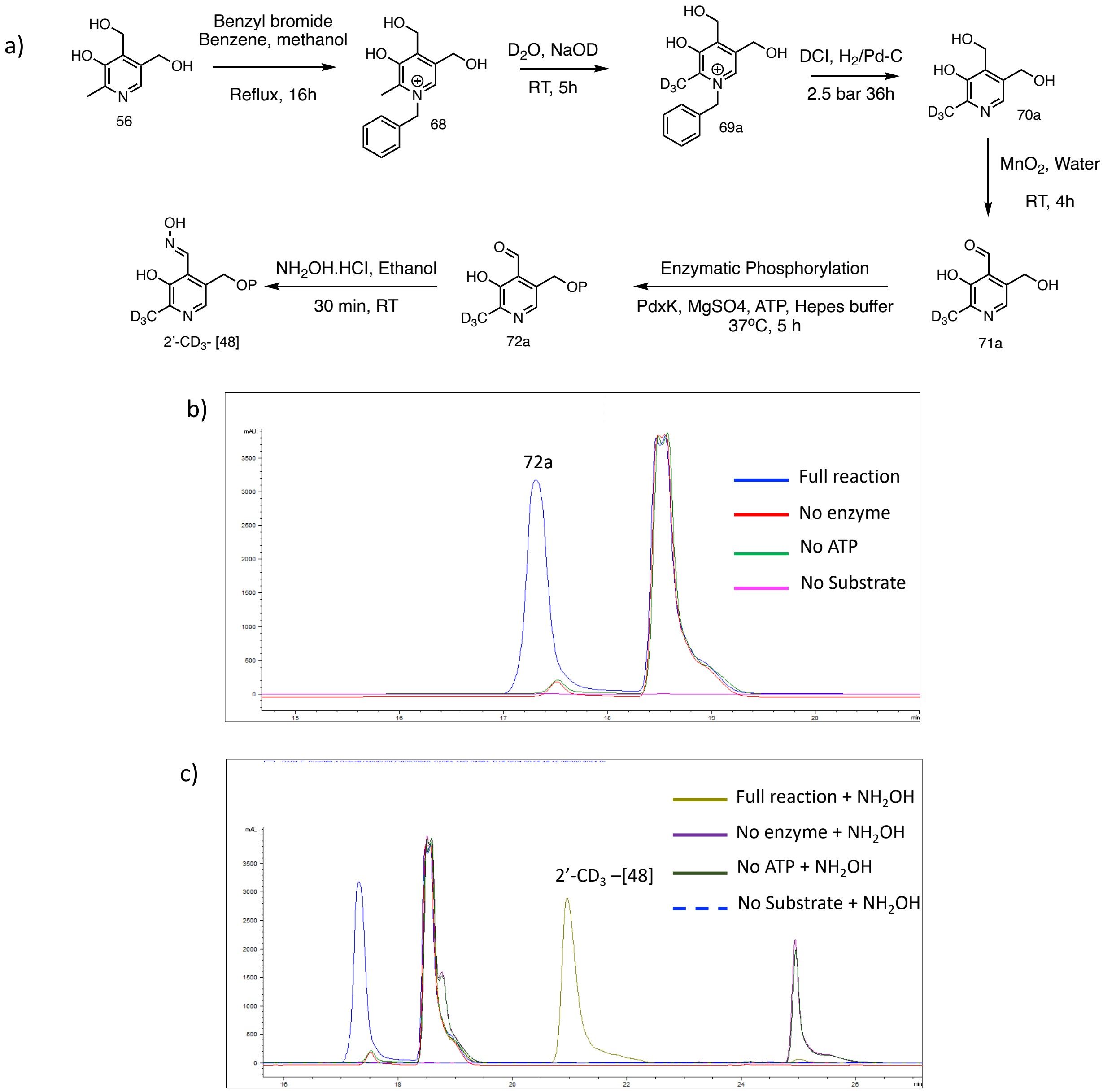

Figure S37. Synthesis of 2'-CD_3-[**48**]. a) Scheme for synthesis of 2'-CD_3-[**48**]. b) HPLC chromatogram at 360 nm showing the enzymatic phosphorylation forming **72a** in the full reaction mixture compared to the indicated controls. c) HPLC chromatogram at 360 nm of the phosphorylation reaction mixture treated with hydroxylamine to form 2'-CD_3-[**48**] in full reaction compared to the indicated controls.

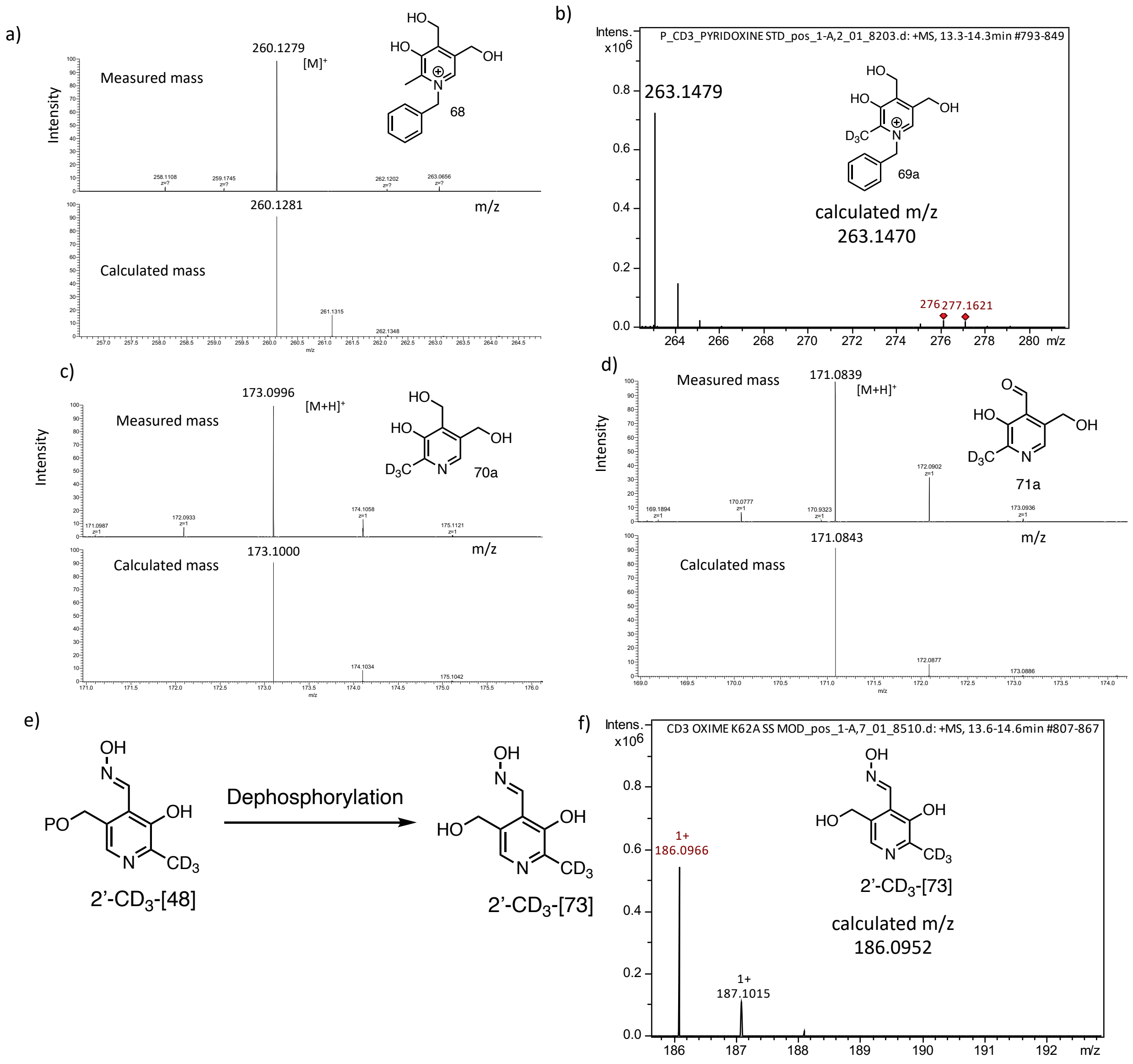

Figure S38. Mass spectra of intermediates in the preparation of 2'-CD_3-[**48**]. a) - d) Mass analysis of **68** to **71a**. e) Dephosphorylation (by CIP treatment) of HPLC purified 2'-CD_3-[**48**]. f) Mass spectrum of 2'-CD_3-[**73**].

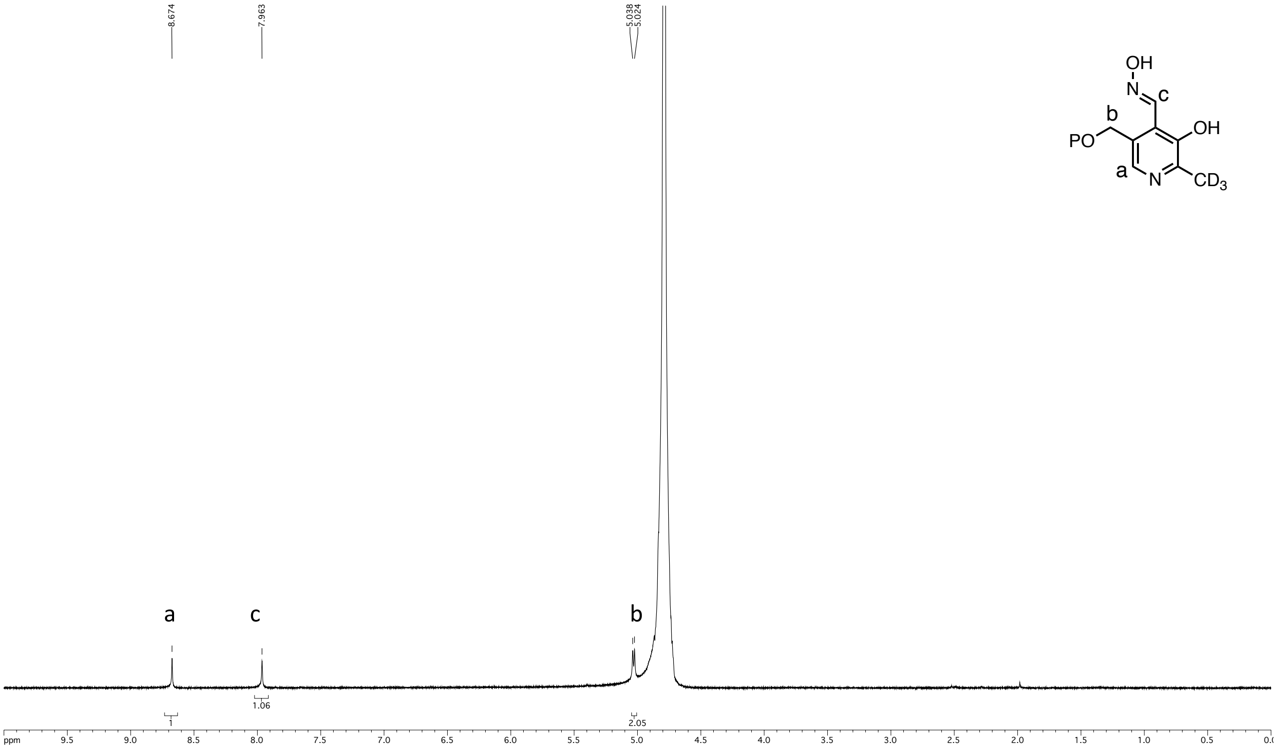

Figure 39. ^{1}H NMR spectrum of 2’-CD_3-[**48**] (400 MHz in D_2O).

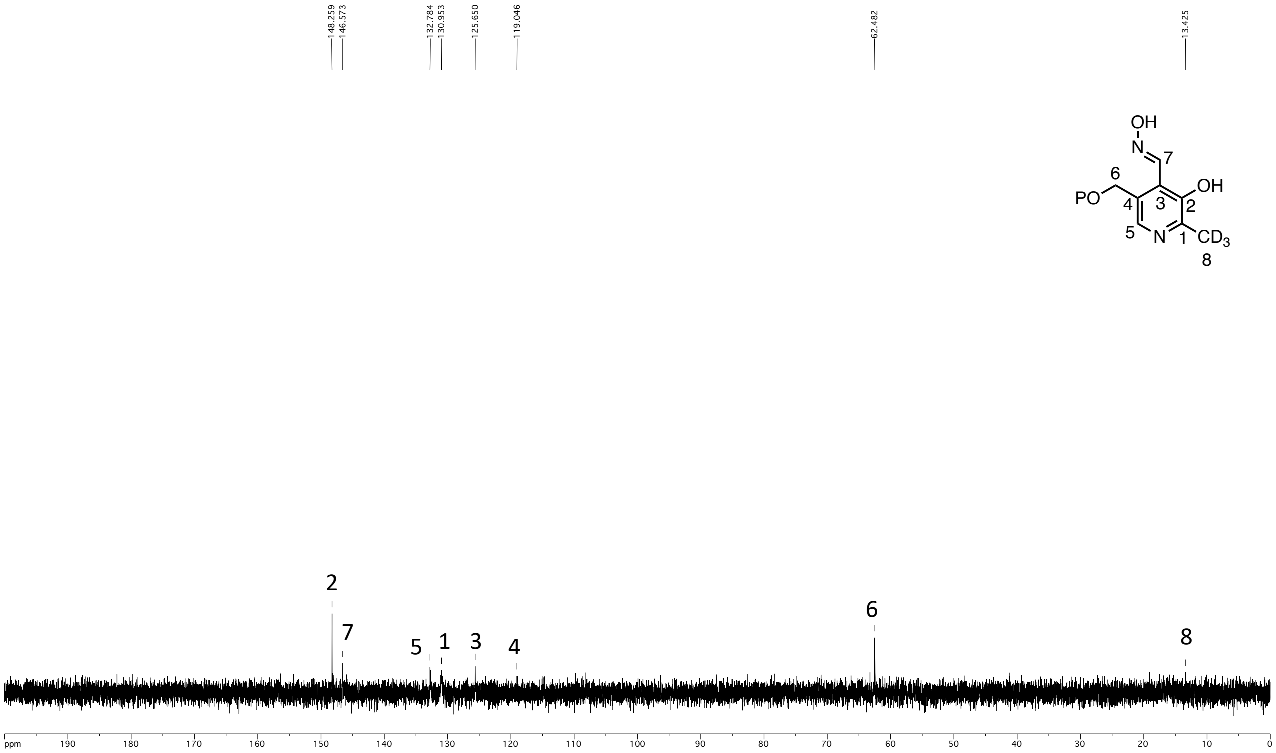

Figure S40. ^{13}C NMR spectrum of 2’-CD_3-[**48**] (100 MHz in D_2O).

References

1. Chan-Huot, M.; Niether, C.; Sharif, S.; Tolstoy, P. M.; Toney, M. D.; Limbach, H.- H. Nmr Studies of the Protonation States of Pyridoxal-5′-Phosphate in Water. *J. Mol. Struct.* **2010**, *976*, 282–289.
2. Lai, R.-Y.; Mondal, A.; Fedoseyenko, D.; Begley, T. P. Mechanistic Studies on the Single-Turnover Yeast Thiamin Pyrimidine Synthase: Characterization of the Inactive Enzyme. *J. Am. Chem. Soc.* **2022**, *144*, 10711-10717.
3. Liu, B.; Wang, R.; Xu, W.; Zhao, G.; Tang, L.; Cheng, X.; Zhou, H. Synthesis and Reaction Mechanism of 3-(4-Methoxyphenylazo) Acrylic Acid. *Drug Discov. Ther.* **2009**, *3*.
4. Lebrasseur, N.; Gagnepain, J.; Ozanne-Beaudenon, A.; Léger, J.-M.; Quideau, S. Efficient Access to Orthoquinols and Their [4+2] Cyclodimers Via Sibx-Mediated Hydroxylative Phenol Dearomatization. *J. Org. Chem.* **2007**, *72*, 6280–6283.
5. Quinn, D. J.; Haun, G. J.; Moura-Letts, G. Direct Synthesis of Nitriles from Aldehydes with Hydroxylamine-O-Sulfonic Acid in Acidic Water. *Tetrahedron Lett.* **2016**, *57*, 3844–3847.
6. Gundry, R. L.; White, M. Y.; Murray, C. I.; Kane, L. A.; Fu, Q.; Stanley, B. A.; Van Eyk, J. E. Preparation of Proteins and Peptides for Mass Spectrometry Analysis in a Bottom-up Proteomics Workflow. *Curr. Protoc. Mol. Biol.* **2010**, *90*, 10.25. 11–10.25. 23.
7. Biemann, K. Contributions of Mass Spectrometry to Peptide and Protein Structure. *Biomed. Environ. Mass Spectrom.* **1988**, *16*, 99–111.
8. ROEPSTORFE, P. Proposal for a Common Nomenclature for Sequence Ions in Mass Spectra of Peptides. *Biomed. Mass Spectrom.* **1984**, *11*, 601–605.
9. Peracchi, A. How (and Why) to Revive a Dead Enzyme: The Power of Chemical Rescue. *Curr. Chem. Biol.* **2008**, *2*, 32–49.
10. Drueckes, P.; Schinzel, R. Activation of E350a Mutant Maltodextrin Phosphorylase by Exogenously Added Acetate. *Protein Engineering, Design and Selection* **1996**, *9*, 701–705.
11. He, Z.; Toney, M. D. Direct Detection and Kinetic Analysis of Covalent Intermediate Formation in the 4-Amino-4-Deoxychorismate Synthase Catalyzed Reaction. *Biochemistry* **2006**, *45*, 5019–5028.
12. Moracci, M.; Trincone, A.; Perugino, G.; Ciaramella, M.; Rossi, M. Restoration of the Activity of Active-Site Mutants of the Hyperthermophilic b-Glycosidase from Sulfolobus Solfataricus: Dependence of the Mechanism on the Action of External Nucleophiles. *Biochemistry* **1998**, *37*, 17262–17270.
13. Williams, D. M.; Wang, D.; Cole, P. A. Chemical Rescue of a Mutant Protein- Tyrosine Kinase. *J. Biol. Chem.* **2000**, *275*, 38127–38130.
14. Proniewicz, L. M.; Bruha, A.; Nakamoto, K.; Kyuno, E.; Kincaid, J. R. Resonance Raman Spectra of Dioxygen Adducts of Cobalt Porphyrin-Imidazole Complexes. Remarkable Spectroscopic Consequences of Hydrogen Bonding of the Coordinated Imidazole and the Lack of an Effect on the Cobalt-Oxygen Linkage. *J. Am. Chem. Soc.* **1989**, *111*, 7050–7056.
15. Coburn, S.; Lin, C.; Schaltenbrand, W.; Mahuren, J. Synthesis of Deuterated Vitamin B6 Compounds. *J. Label. Compd. Radiopharm.* **1982**, *19*, 703–716.

16. Heyl, D.; Luz, E.; Harris, S. A.; Folkers, K. Phosphates of the Vitamin B6 Group. I. The Structure of Codecarboxylase. *J. Am. Chem. Soc.* **1951**, *73*, 3430–3433.
17. Weeks, K. L.; Rutkowski, K. R.; Loyola, A. A. M.; Boyce, G. R. Utilization of Pyridoxal Acetal Salts as Water-Triggered, Slow-Release Pro-Fragrances. *New J. Chem.* **2018**, *42*, 15538-15540.
18. Pocker, A. Synthesis of 2-nor-2-Formylpyridoxal 5'-Phosphate, a Bifunctional Reagent Specific for the Cofactor Site in Proteins. *J. Org. Chem.* **1973**, *38*, 4295–4299
19. Dale, T. J.; Sather, A. C.; Rebek Jr, J. Synthesis of Novel Aryl-1, 2-Oxazoles from Ortho-Hydroxyaryloximes. *Tetrahedron Letters* **2009**, *50*, 6173–6175.
20. Aitken, R. A.; Raut, S. V. A Convenient Mild Two-Step Conversion of Imines to Secondary and Tertiary Amides. *Synlett* **1991**, *1991*, 189–190.
